# Supplementary figures and images for: Optimized criteria for locomotion-based healthspan evaluation in C. elegans using the WorMotel system
Source: PLoS One. 2020 Mar 3;15(3):e0229583. doi: 10.1371/journal.pone.0229583 (PMC7053758; doi:10.1371/journal.pone.0229583)

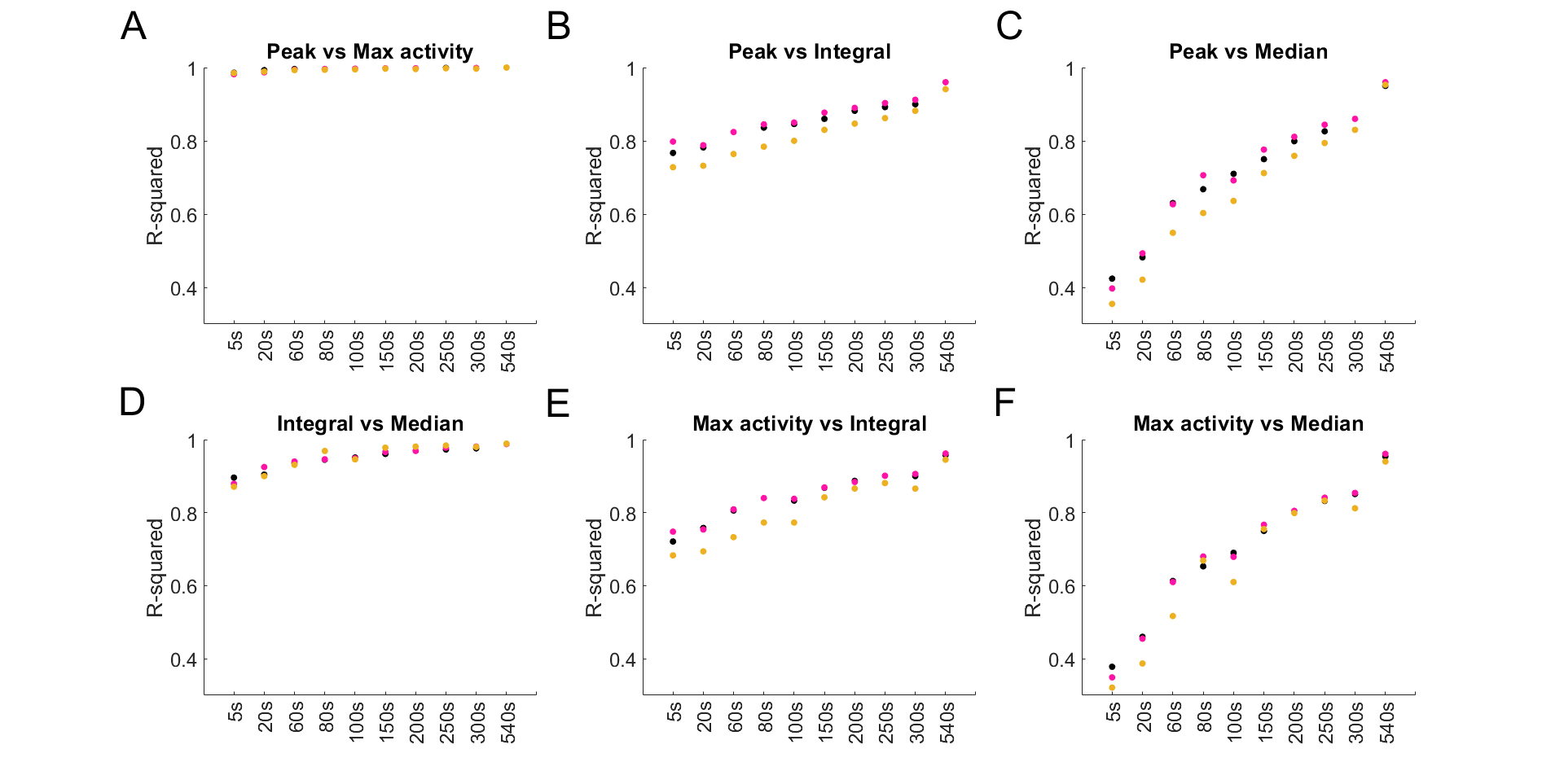

Supplement: S1 Fig — Activity calculated by using the peak values correlates perfectly with maximal activity values (99th percentile) at any time interval (A), whereas correlation with integral (B) and median (C) values is time interval dependent. (D) Integral values, on the contrary, correlate well with median values, but neither of these (E integral, F median) escape the weaker and interval-dependent correlation with maximal activity. These data suggest that only two interpretations of the activity profile are made by determination of peak/maximal and median/integral daily activities. Time interval dependence of the correlations in B, C, E and F is easily explained by the higher sensitivity of median/integral values to the time interval between analyzed images. (TIF) [file pone.0229583.s001.tif]

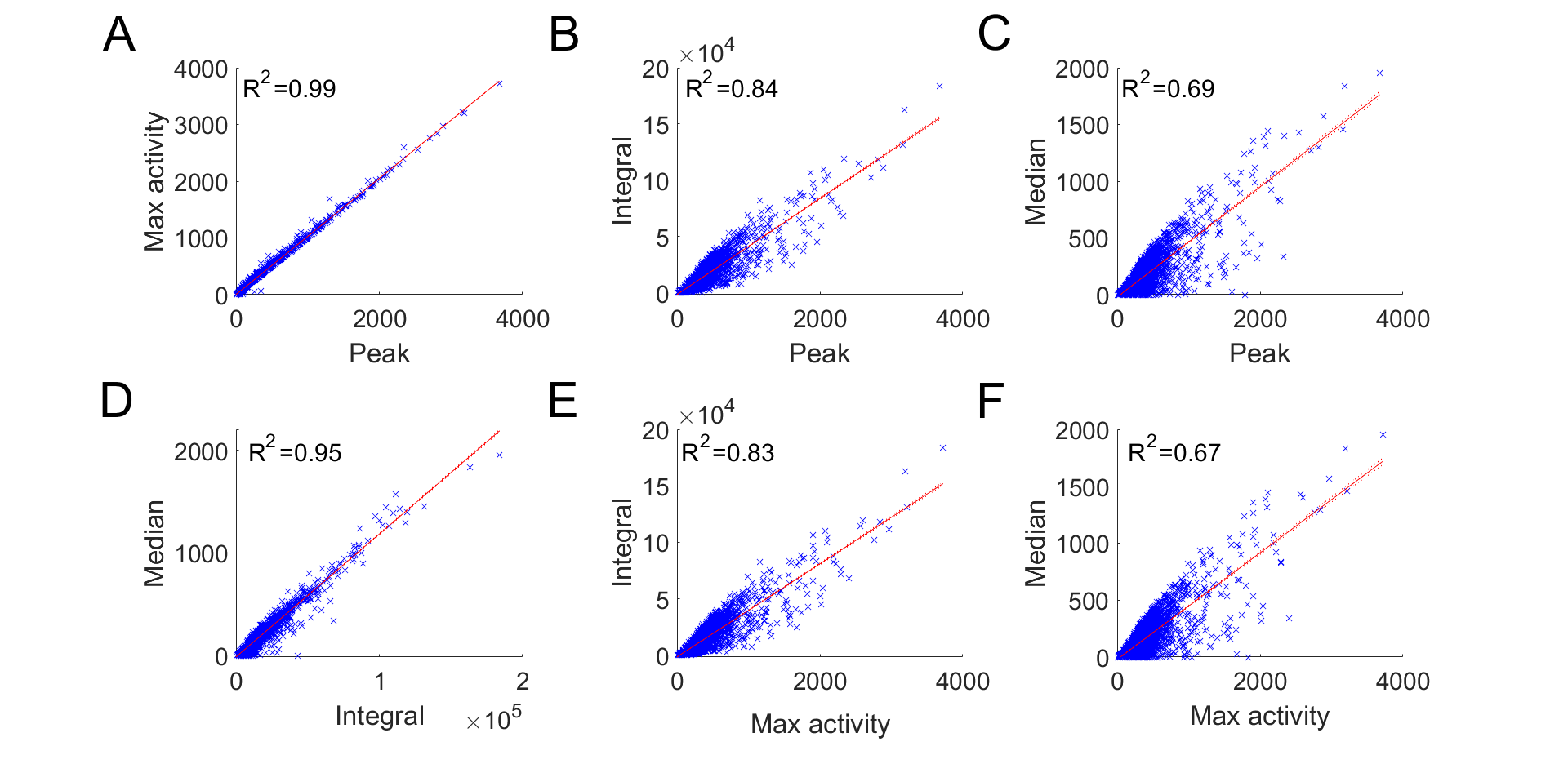

Supplement: S2 Fig — Activity was calculated based on the different parameters for each worm on each day, independent of genotype. (A) Activity calculated by using the peak values correlates perfectly with maximal activity values (99th percentile), whereas correlation with (B) integral and (C) median values is less pronounced. (D) Integral values, on the contrary, correlate well with median values, but both (E integral, F median) show a weaker correlation with the maximal value. (TIF) [file pone.0229583.s002.tif]

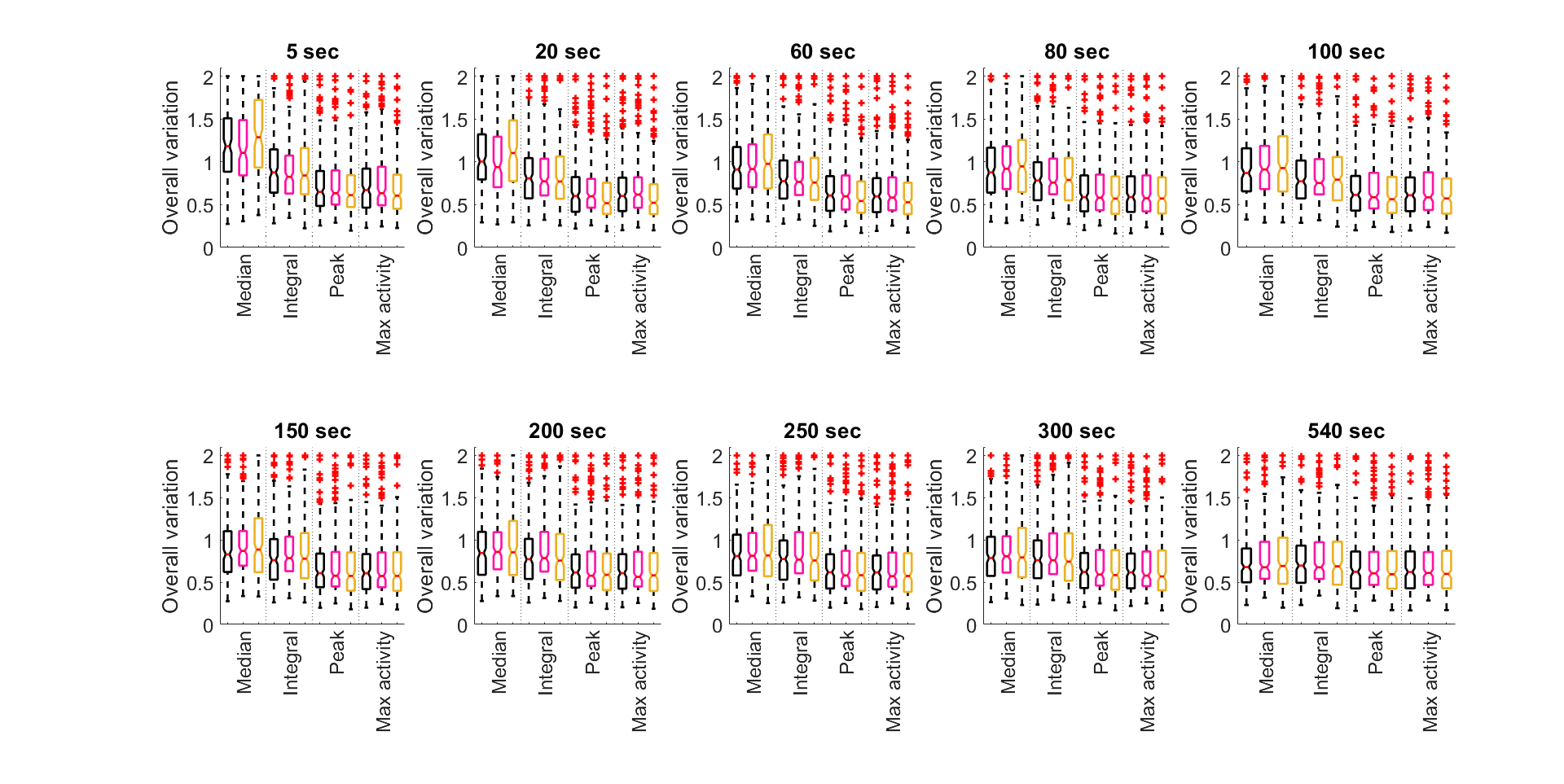

Supplement: S3 Fig — For each individual, day-to-day variation was calculated as stated in the main text. Box values: Q1-2-3, whiskers: +/–2.7σ. (TIF) [file pone.0229583.s003.tif]

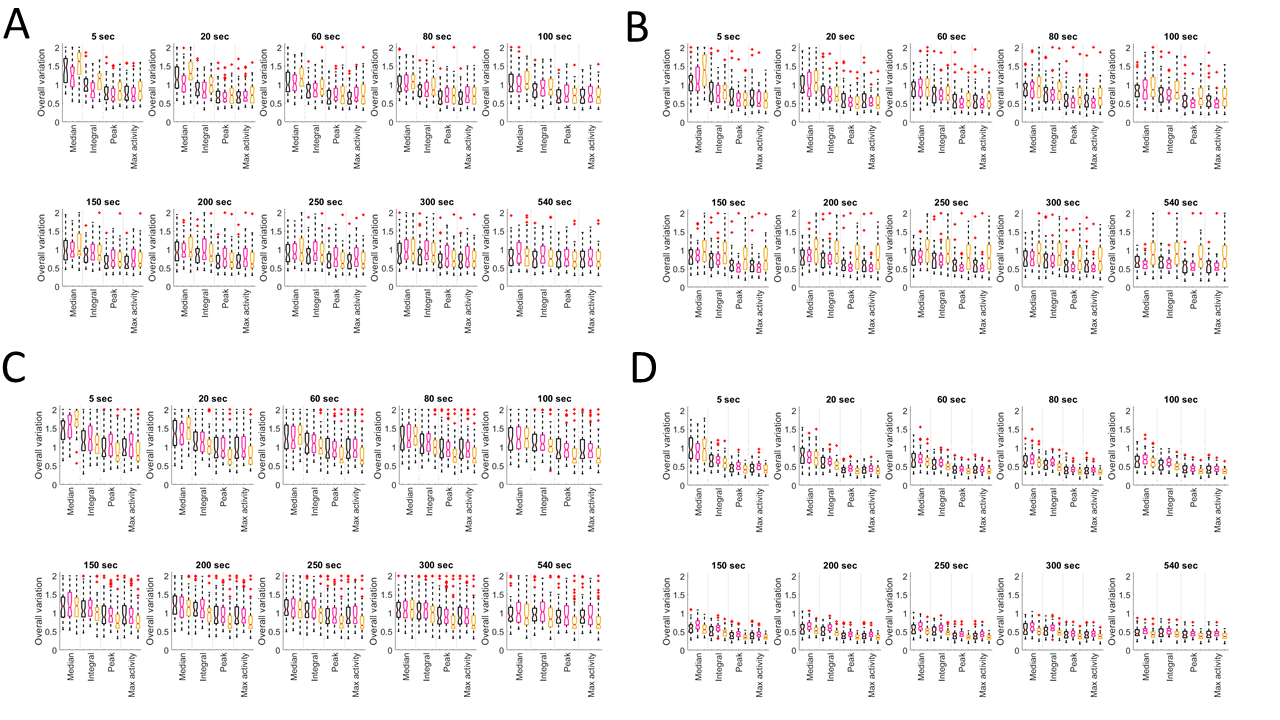

Supplement: S4 Fig — Distributions of overall variation based on daily median, integrated, peak or maximal activity for control (black), daf-2 (pink) or daf-16 (yellow) RNAi-treated populations across all experiments (A Exp I; B Exp II; C Exp III; D Exp IV) follow the same trends as pooled data (Fig 5). For each individual, day-to-day variation was calculated as stated in the main text. Box values: Q1-2-3, whiskers: +/–2.7σ. (TIF) [file pone.0229583.s004.tif]

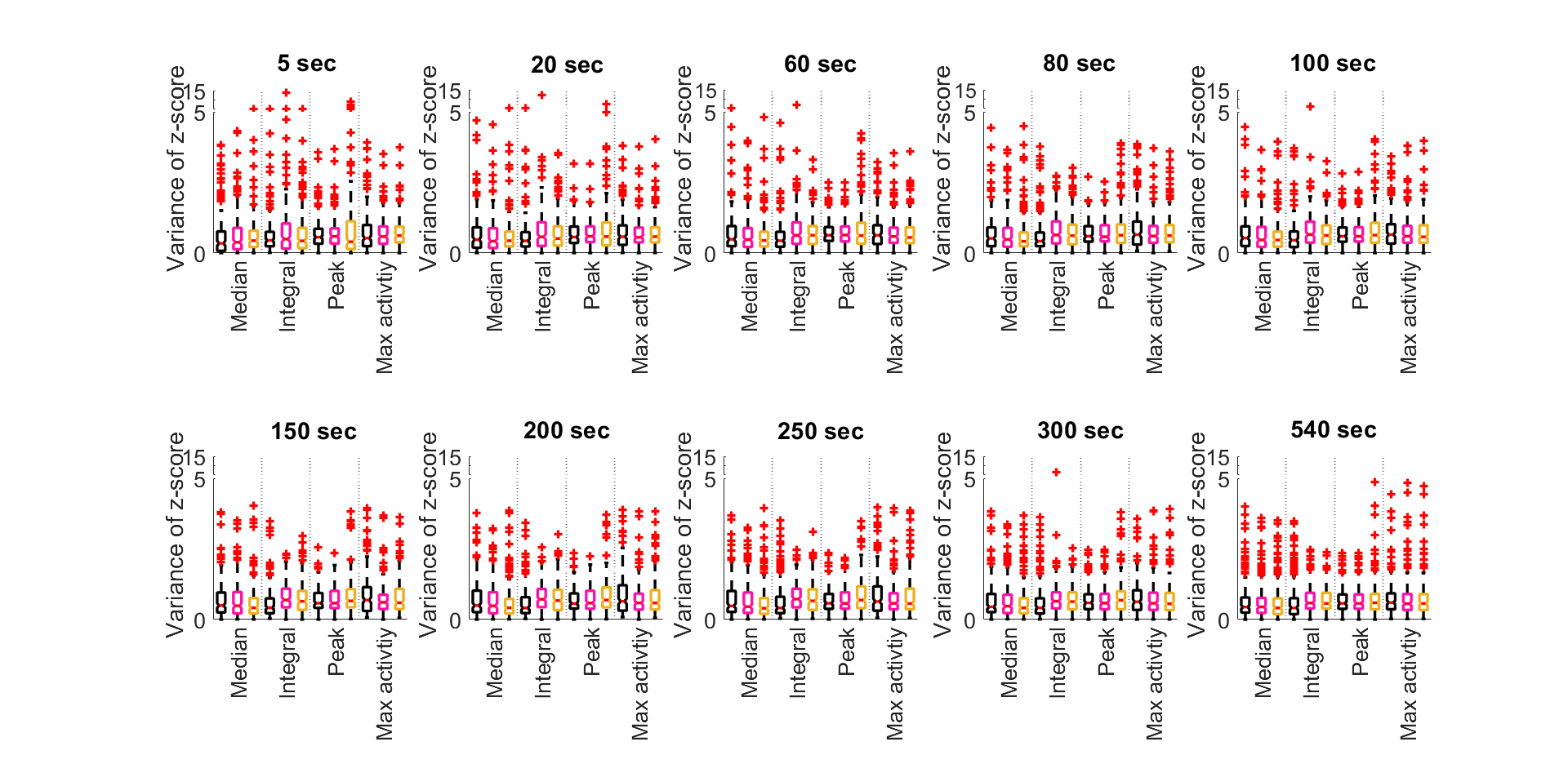

Supplement: S5 Fig — For each individual, day-to-day variation was calculated as stated in the main text. Box values: Q1-2-3, whiskers: +/–2.7σ. (TIF) [file pone.0229583.s005.tif]

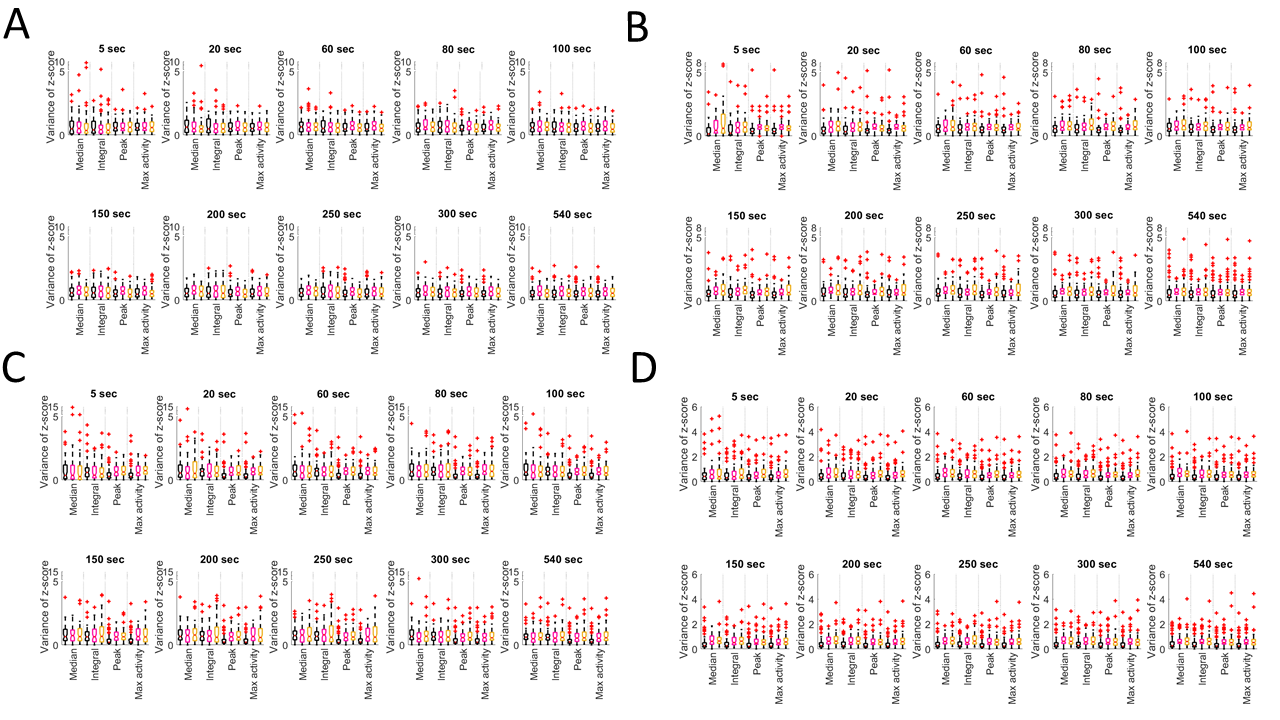

Supplement: S6 Fig — Variance in Z-score based on individual plates is similar for all activity parameters at all time intervals, as is clear from the distributions based on daily median, peak, maximal or integrated activity for control (black), daf-2 (pink) or daf-16 (yellow) RNAi-treated populations across all experiments (A Exp I; B Exp II; C Exp III; D Exp IV). For each individual, day-to-day variation was calculated as stated in the main text. Box values: Q1-2-3, whiskers: +/–2.7σ. (TIF) [file pone.0229583.s006.tif]

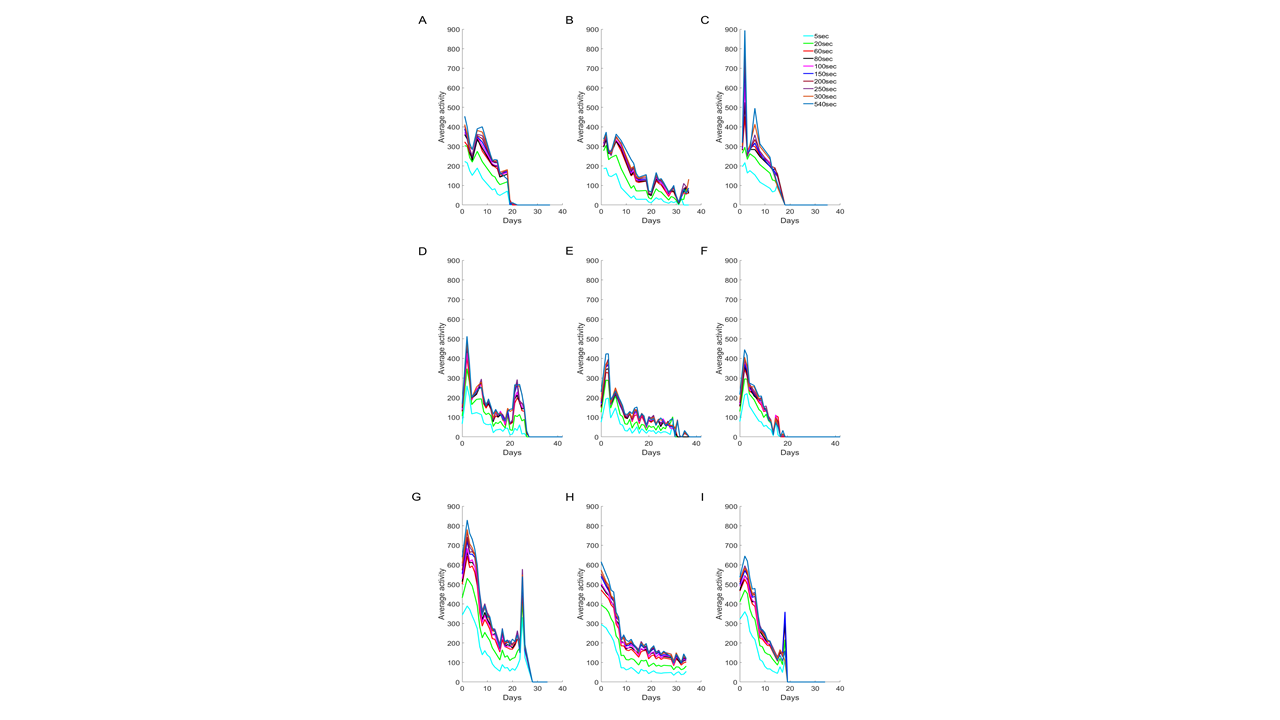

Supplement: S7 Fig — Average survivor activity for control, daf-2 and daf-16 RNAi-treated populations for (A-C) Exp II, (D-F) Exp III and (G-I) Exp IV. Longer time intervals (≥60s) provide more accurate measurements, this is especially important in late phases of life. (TIF) [file pone.0229583.s007.tif]

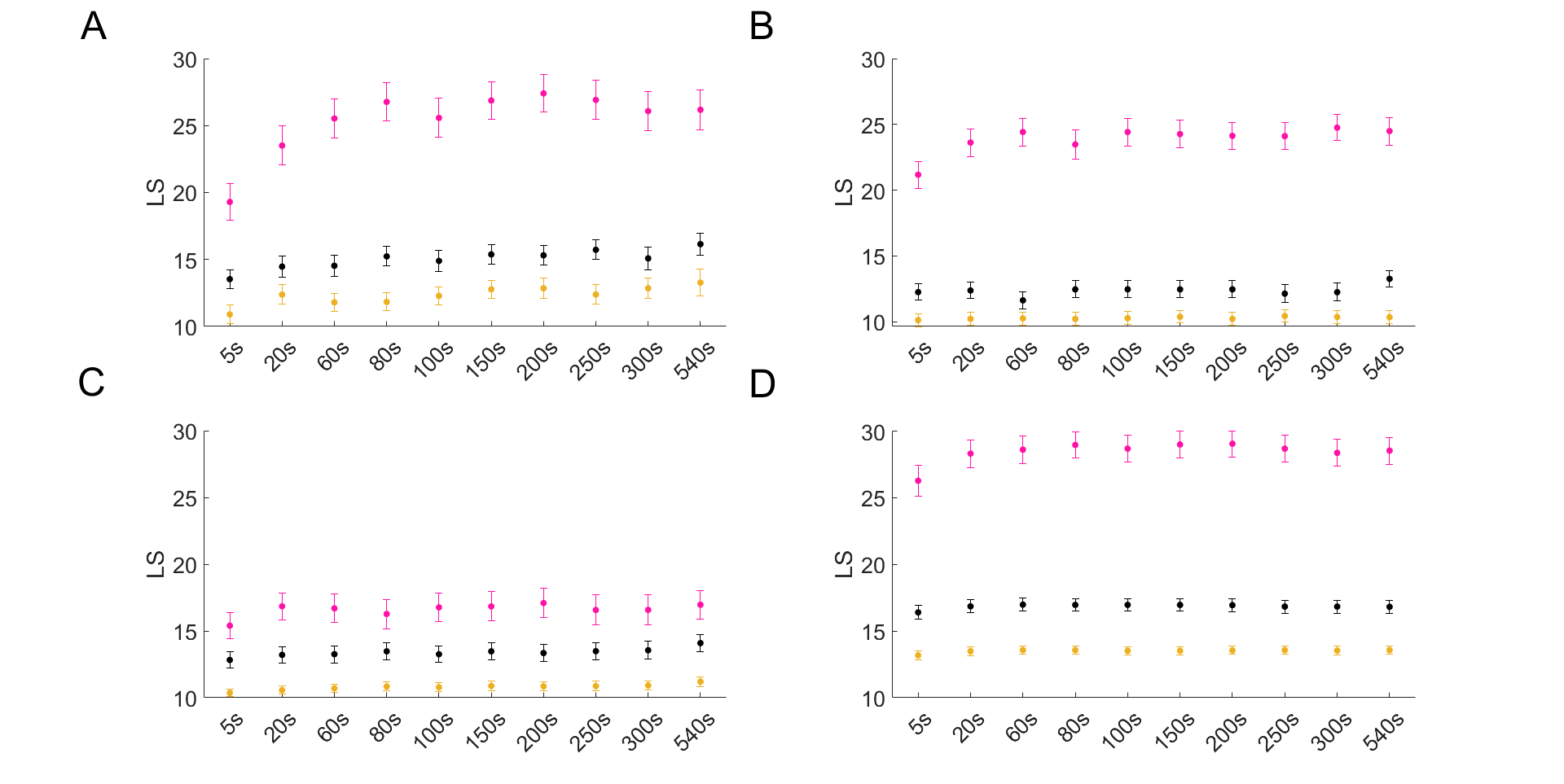

Supplement: S8 Fig — Mean lifespan (error bars: standard error of mean) was calculated for different time intervals for (A) Exp I, (B) Exp II, (C) Exp III and (D) Exp IV. The choice of time interval does not affect the calculation of lifespan of control (black) and daf-16 RNAi-treated (yellow) populations but does affect lifespan decisions made for the long-lived daf-2 RNAi-treated (pink) populations. (TIF) [file pone.0229583.s008.tif]

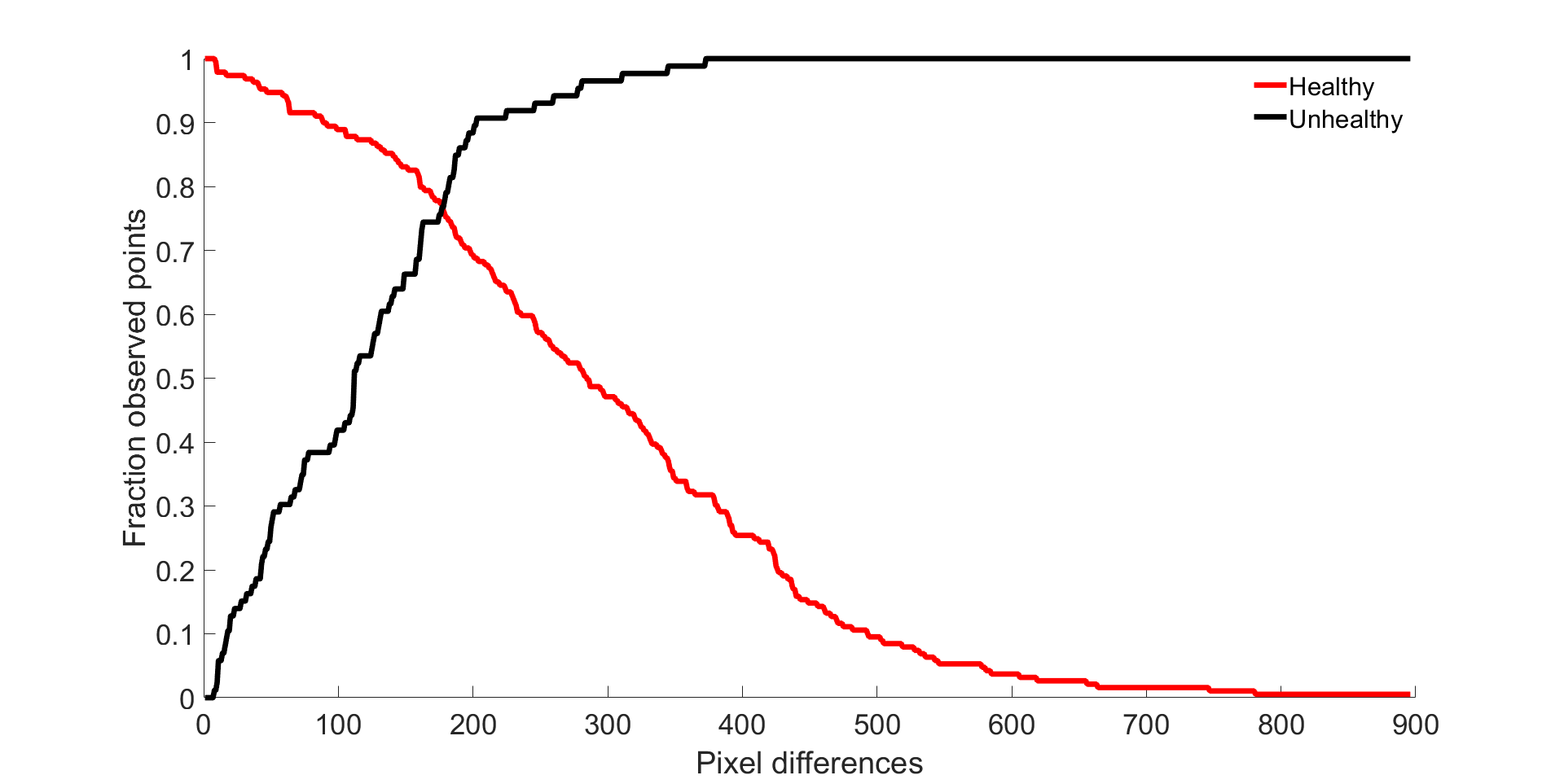

Supplement: S9 Fig — Determination of a threshold that maximizes the number of truly healthy worms in healthy (very fast—fast—medium fast) categories, while maximizing the number of truly unhealthy worms in the unhealthy categories (slow—inactive) led to a threshold value of 177 pixels changed. Black line: fraction of animals in the 'slow' category with a pixel difference value < x-axis value; red line: fraction of animals in the ‘very fast’, ‘fast’ and 'medium fast' category with a pixel difference value >x-axis value. (TIF) [file pone.0229583.s009.tif]

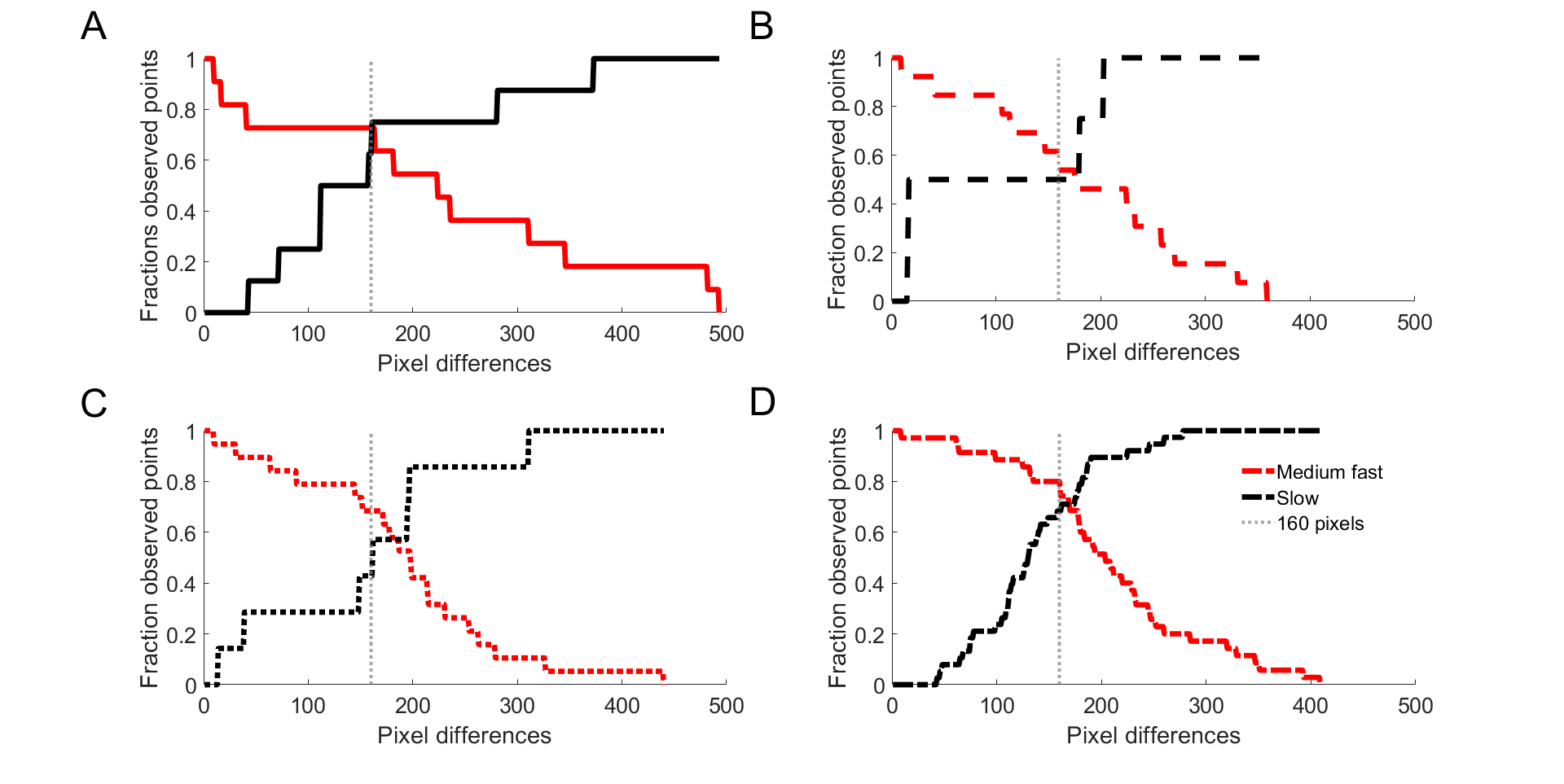

Supplement: S10 Fig — Threshold determination on individual plate level is very similar to pooled data, with cumulative curves of from medium fast and slow worms intersecting at approximately 160 pixel differences for (A Exp I; B Exp II; C Exp III; D Exp IV). (TIF) [file pone.0229583.s010.tif]

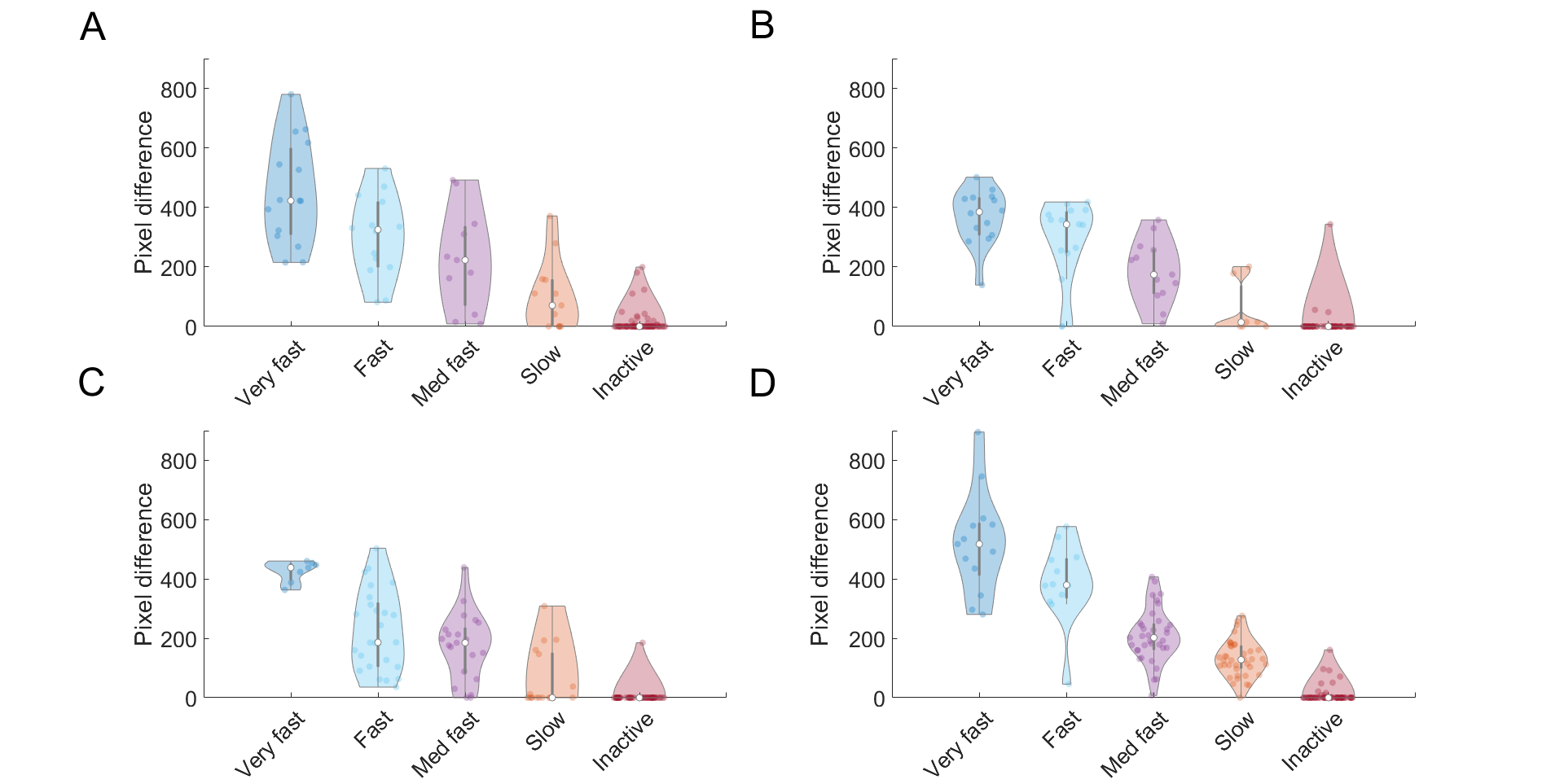

Supplement: S11 Fig — In general, pixel differences below 160 belong to categories 4 and 5 for A Exp I; B Exp II; C Exp III; D Exp IV. (TIF) [file pone.0229583.s011.tif]

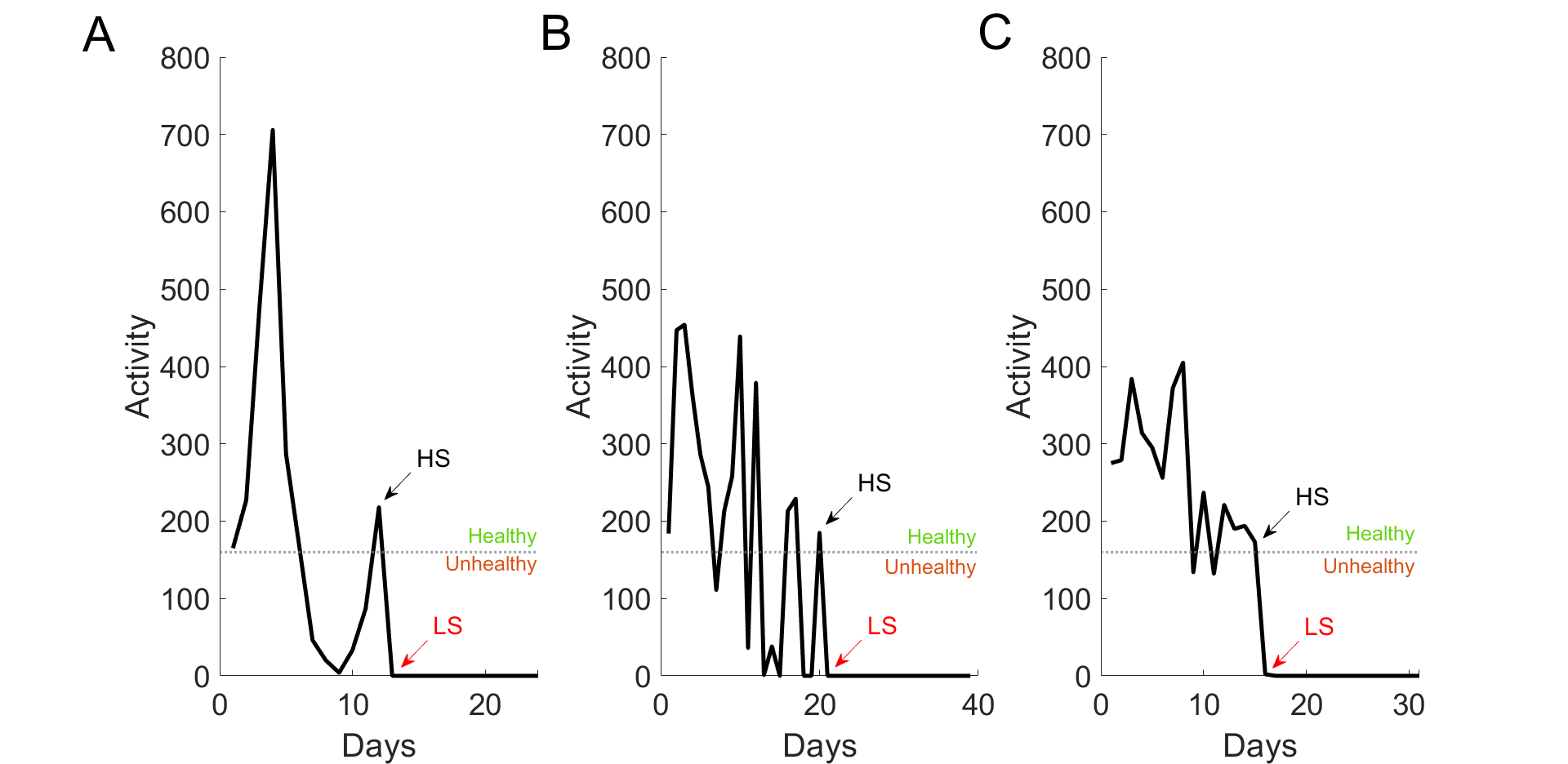

Supplement: S12 Fig — We plotted the activity profile of three individual wild-type worms whose HS was 1, but whose TDH (A) strongly, (B) moderately or (C) slightly deviated from HS. Activity profile of (A) shows a flare of activity in the last day of life, resulting in a misleadingly high HS. TDH of (B) nears HS more than in case of (A), however, fluctuations in the activity profile of this worm indicate that TDH has a better representation of the animal’s health. (C) TDH deviates only two days from the quantified HS, nevertheless, leads to a better approximation of health. All three worms visually indicate that HR (~TDH) is a more accurate quantification of observed health than HSR (~HS). (TIF) [file pone.0229583.s012.tif]

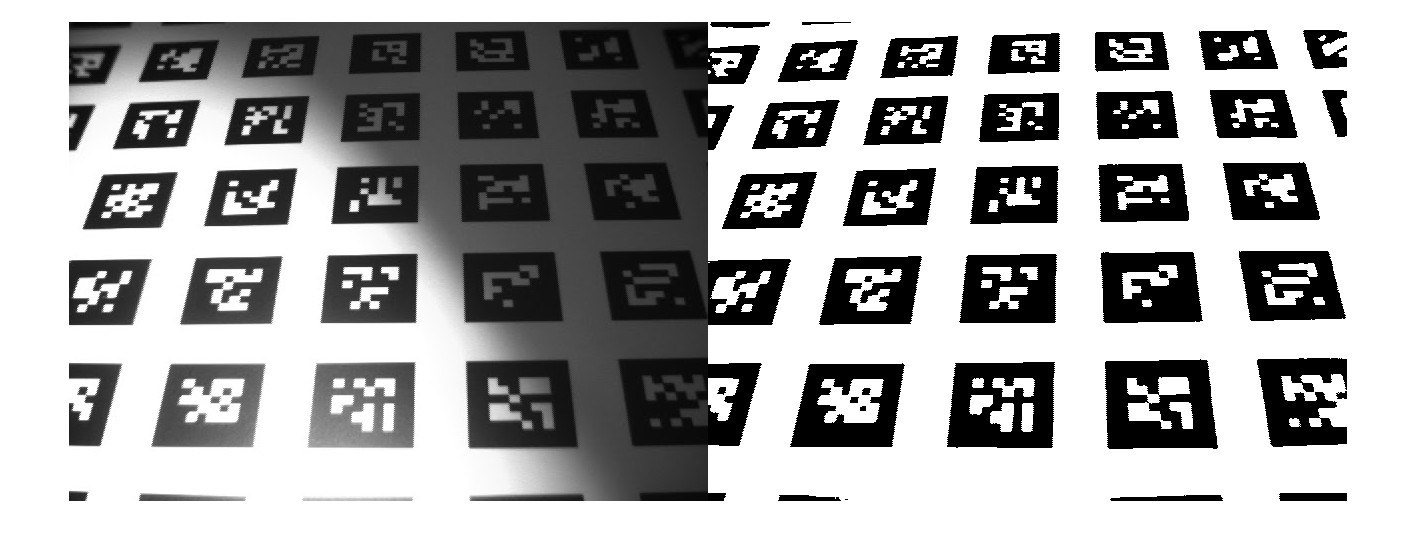

Supplement: S2 File — This includes relevant scripts (See Tutorial). (ZIP) [file pone.0229583.s020.zip › S Tutorial/Step1_Imageprocessing/Dependencies/bradley/bradley/bradley.png]
