# Supplementary material for: Optimized criteria for locomotion-based healthspan evaluation in C. elegans using the WorMotel system
Source: PLoS One. 2020 Mar 3;15(3):e0229583. doi: 10.1371/journal.pone.0229583 (PMC7053758; doi:10.1371/journal.pone.0229583)
Supplement: S2 File — This includes relevant scripts (See Tutorial). (ZIP) [file pone.0229583.s020.zip › S Tutorial/Step1_Imageprocessing/Dependencies/NetGraphics/Pan and Zoom Very Large Images - CodeProject.pdf]

11,981,699 members (65,725 online)

JungTemp 861 Sign out

Merry Christmas
CODE PROJECT®
For those who code

articles Q&A forums lounge

Search for articles, questions, tips

Pan and Zoom Very Large Images

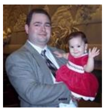

Anthony Queen, 31 Oct 2009 Ms-PL

4.84 (134 votes)

Rate:

Smoothly panning and zooming very large images can be a challenge. Here's a control, with source code, that demonstrates one way of overcoming this challenge, as well as a few "Extra" image processing features.

Is your email address OK? You are signed up for our newsletters but your email address is either unconfirmed, or has not been reconfirmed in a long time. Please click **here** to have a confirmation email sent so we can confirm your email address and start sending you newsletters again. Alternatively, you can **update your subscriptions**.

Download source code/executable (.NET 2.0) - 69.79 KB

Note: This was written against .NET 2.0, then manually converted to .NET 1.1.

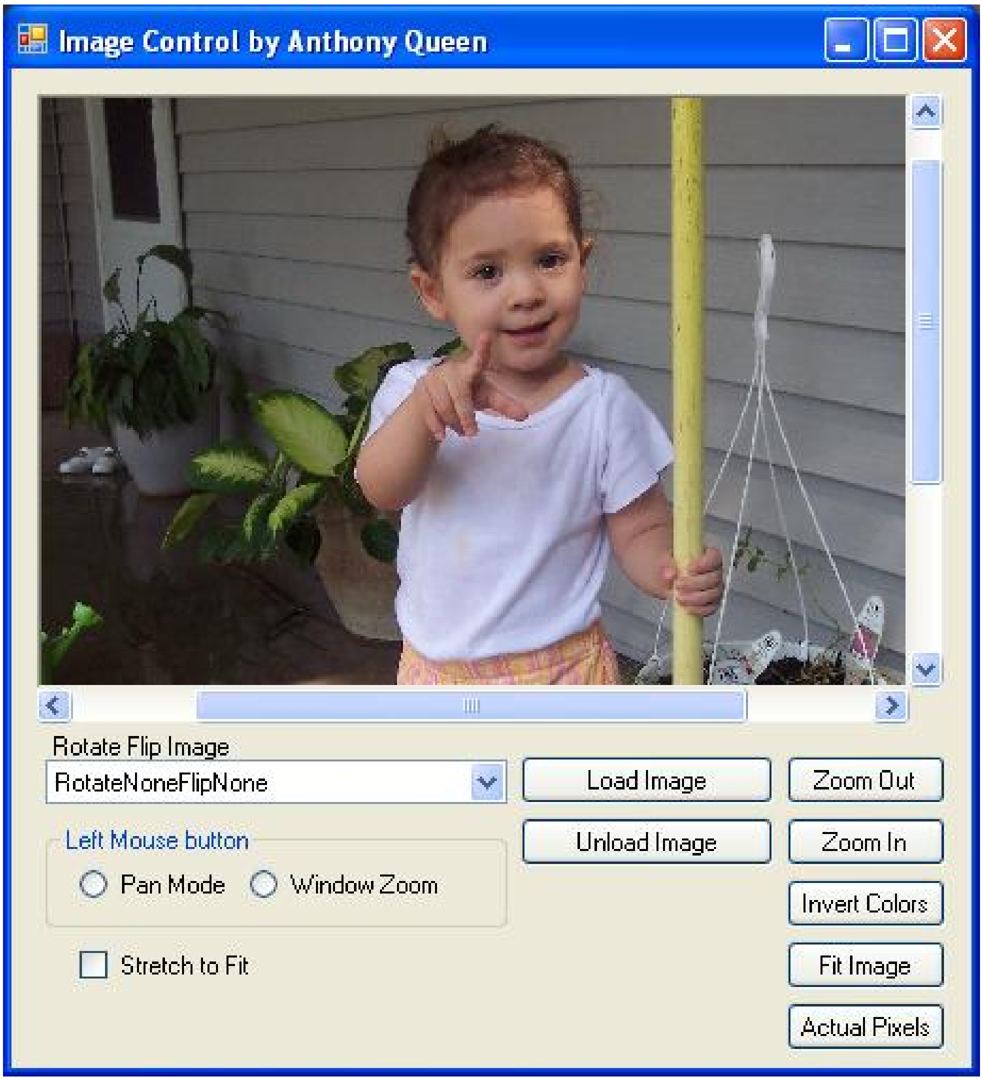

Introduction

I recently wrote an article showing a simple method for panning an image. The code worked very well for small to moderate sized images. However, when using very large images, the performance degraded significantly.

That article used a picture box within a panel, and used the auto scroll functionality of the panel to perform scrolling. I received quite a bit of feedback indicating the need for a version that could handle **very** large images and still pan very smoothly. I also received requests for ideas on how to zoom the image in and out. So, I got to work.

What I came up with is a control that could smoothly pan super-sized images, and also provided zoom functionality. My tests were with a 49MB GIF (7000 x 7000). The performance was very smooth. Of course, the control works equally as well with small images. The control is demonstrated in the included sample project.

This custom control does **not** use a picture box, nor does it inherit from one. Neither is there a panel or any "auto-scrolling". This is very different and very much a better way of panning an image (in my opinion). An added benefit to this example is the ability to zoom the image **without** resizing a picture box (which can get quite large in memory).

How It Works

1. Only paints the part of the image currently visible.
2. Double-buffering provides flicker free panning.
3. GDI+ automatically scales the image for us.

Public Properties

- `Public Property PanButton() As System.Windows.Forms.MouseButtons`
- `Public Property ZoomOnMouseWheel() As Boolean`
- `Public Property ZoomFactor() As Double`
- `Public Property Origin() As System.Drawing.Point`

Public Shadows

- `Public Shadows Property Image() As System.Drawing.Image`
- `Public Shadows Property initialimage() As System.Drawing.Image`

Public Methods

- `Public Sub ShowActualSize()`
- `Public Sub ResetImage()`

Using the control is as simple as using a standard `PictureBox`. First, drop the control on a form, then when you need to show an image, you can do it this way:

Hide Copy Code

```
Dim bmp As New Bitmap("Image.jpg")
Me.ImageViewer1.Image = bmp
```

Don't forget to change the filename!

It is important to note: If you are working with very large images, you should not pre-load them in the designer. This seriously bloats the project, and can result in "Out of Memory" issues. Instead, load your images during run-time.

Default Behavior

- **Panning the image:** Click and hold the left mouse button while the cursor is over the image. Then, simply move your mouse around, with the button still depressed.
- **Zooming:** Make sure the control has focus (click the image). Then, use your mouse wheel to zoom in and out.

Customized Usage

You can tell the control what button to use for panning, with the "`PanButton`" property. You can turn off the default zooming by setting the `ZoomOnMouseWheel` property to `False`.

You can manually set the zoom factor so you could implement your own zoom functionality (i.e., using a slider, or buttons).

You can move the image around programmatically by setting the origin. The origin property gets or sets the coordinates of the top left corner of the viewable window in relation to the original image. For example, if you wanted to see the bottom right corner of an image with a size of 5000 x 5000, and your viewable control size was 500 pixels x 500 pixels, you could set the origin to 4500, 4500. This assumes, of course, that you have a zoom factor of 1 (not zoomed in or out).

You could catch the paint event of the control and overlay your own graphics. Just be careful to take the zoom factor into consideration if you need to draw at precise coordinates in relation to the original image.

Scrollbars

Due to popular demand, scrollbars have now been implemented.

Double Buffering

Double buffering is accomplished by setting the control styles in the constructor as such:

Hide Copy Code

```
Public Sub New()
     MyBase.New()
     'This call is required by the Windows Form Designer.
     InitializeComponent()
     'Add any initialization after the InitializeComponent() call
     Me.SetStyle(ControlStyles.AllPaintingInWmPaint, True)
     Me.SetStyle(ControlStyles.DoubleBuffer, True)
End Sub
```

Just In Time Painting?

Well, sort of. While we do have a copy of the image in memory, we only paint the area currently viewable.

Hide Copy Code

```
Protected Overrides Sub OnPaint(ByVal e As PaintEventArgs)
     e.Graphics.Clear(Me.BackColor)
     DrawImage(e.Graphics)
     MyBase.OnPaint(e)
End Sub

Protected Overrides Sub OnSizeChanged(ByVal e As EventArgs)
     DestRect = New System.Drawing.Rectangle(0, 0, _
                    ClientSize.Width, ClientSize.Height)
     MyBase.OnSizeChanged(e)
End Sub

Private Sub DrawImage(ByRef g As Graphics)
     If m_OriginalImage Is Nothing Then Exit Sub
     SrcRect = New System.Drawing.Rectangle(m_Origin.X, m_Origin.Y, _
                     ClientSize.Width / m_ZoomFactor, _
                     ClientSize.Height / m_ZoomFactor)
     g.DrawImage(m_OriginalImage, DestRect, SrcRect, GraphicsUnit.Pixel)
End Sub
```

Note that we are taking the current zoom factor into consideration when drawing. By using the `DrawImage` method of the `Graphics` object, GDI will scale the image from the source area to fit the destination area.

Panning the Image

The code for panning the image and keeping the zoom factor in mind, is as follows:

Hide Shrink ▲ Copy Code

```
Private Sub ImageViewer_MouseMove(ByVal sender As Object, _
        ByVal e As System.Windows.Forms.MouseEventArgs) _
        Handles MyBase.MouseMove

     'Make sure we are panning on the correct mouse button
     If e.Button = m_MouseButtons Then
          Dim DeltaX As Integer = m_PanStartPoint.X - e.X
          Dim DeltaY As Integer = m_PanStartPoint.Y - e.Y

          'Set the origin of the new image
          m_Origin.X = m_Origin.X + (DeltaX / m_ZoomFactor)
          m_Origin.Y = m_Origin.Y + (DeltaY / m_ZoomFactor)
```

```
            'Make sure we don't go out of bounds
            If m_Origin.X < 0 Then m_Origin.X = 0
            If m_Origin.Y < 0 Then m_Origin.Y = 0

            If m_Origin.X > m_OriginalImage.Width - _
                           (ClientSize.Width / m_ZoomFactor) Then
                 m_Origin.X = _m_OriginalImage.Width - _
                              (ClientSize.Width / m_ZoomFactor)
            End If
            If m_Origin.Y > m_OriginalImage.Height - _
                             (ClientSize.Height / m_ZoomFactor) Then
                 m_Origin.Y = m_OriginalImage.Height - _
                             (ClientSize.Height / m_ZoomFactor)
            End If

            If m_Origin.X < 0 Then m_Origin.X = 0
            If m_Origin.Y < 0 Then m_Origin.Y = 0

            'reset the startpoints
            m_PanStartPoint.X = e.X
            m_PanStartPoint.Y = e.Y

            'Force a paint
            Me.Invalidate()
        End If
    End Sub
```

Conclusion

Many of the concepts used within this example project are worthy of their own discrete articles. Therefore, I didn't go into any great detail about what double buffering is, nor did I dive into the intricacies of GDI+ in .NET. However, I hope that I have adequately covered the basics of how this control works, as well as how you can use it.

Please Note...

This is by no means meant to be a complete solution, nor is this code "production-ready". Then too, there are usually many ways to solve a problem; this is one. Hopefully, though, this sample has proven beneficial in some way. Perhaps, this article has given you a great idea about how to do this a better way, or an idea for expanding what is presented here. Great! That's why I wrote it. Please feel free to leave some feedback. Let me know how it went for you. If you do have an idea on how to improve this example or this article, please let me know that too.

P.S.: Don't forget to vote! If you don't have an account, make one!

Revisions and Bug Fixes ...

- **02/04/2007**
  - Added scrollbar functionality
  - Fixed null image bug
  - Fixed memory leak
  - Implemented several performance improving suggestions
  - Added ability to invert colors
  - Added ability to stretch image or set to actual pixels
  - Removed the hard coded image file and added dialogue box to test harness
- **02/06/2007**
  - Added a .NET 1.1 version

- **30/10/2009**
  - Deleted the .NET 1.1 zip file

TO DO

1. Change `Point`s to `PointF` and `Rectangle`s to `RectangleF` to allow finer panning and scrolling when zoomed in very tight
2. Update the article to dissect the app and explain why it works the way it does
3. Update the code presented in the article

Thanks for your patience!

License

This article, along with any associated source code and files, is licensed under The Microsoft Public License (Ms-PL)

Share

EMAIL TWITTER

About the Author

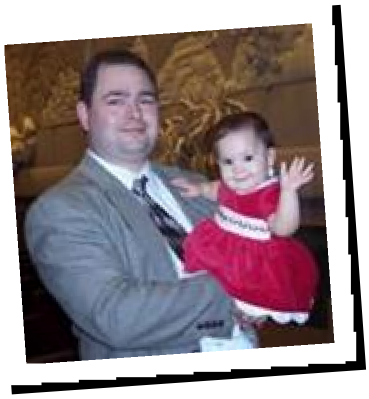

Anthony Queen

Software Developer (Senior)
United States

I started my career in software development back in 2000. Prior to that, I made my living as a detail drafter. My true start in programming, though, goes back much further. I first started learning to program when I was about 10 years old. It was back in '82 that I wrote my first application. It was a simple calculator program written on a TRS-80 that my uncle had. Since then, I've programmed in Basic, QuickBasic, Pascal, C++, VB 6, VB.NET, Java, HTML, and C #. I have a very diverse background. I've worked and written software for several types of companies, including manufacturing, engineering, and finance. I've had the opportunity to design and maintain a few enterprise level databases, I've written applications to run on windows CE, in a wireless manufacturing environment. I've also had opportunities to teach OOP methodologies, and design patterns. I thoroughly enjoy what I do, and my only regret is that I didn't start sooner.

You may also be interested in...

A WPF Custom Control for

Synchronizing Large

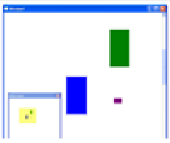

Zooming and Panning

Engineering Source Code Repositories

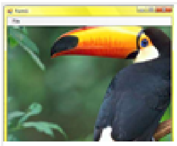

Image Target Zoom (Pan Zoom)

Barcodescanner with webcam on Intel® Edison

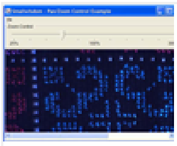

ImageBox Control with Zoom/Pan Capability

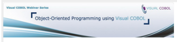

COBOL programmers: Skill up and save time...

Comments and Discussions

Add a Comment or Question

Search Comments Go

First Prev Next

Re: Large image and time loading
splater 15-Jan-08 11:36

Re: Large image and time loading
Anthony Queen 16-Jan-08 13:49

Retreiving Coordonates
splater **23-Nov-07 4:07**

Re: Retreiving Coordonates
splater 15-Jan-08 5:38

Re: Retreiving Coordonates
jronecube 29-Feb-08 14:19

Re: Retreiving Coordonates
splater 3-Mar-08 3:49

Re: Retreiving Coordonates
jronecube 3-Mar-08 14:41

Re: Retreiving Coordonates
splater 4-Mar-08 4:26

inserting more than one image in the control.
Holochris **17-Oct-07 1:00**

Re: inserting more than one image in the control.
Anthony Queen 24-Oct-07 20:24

problem on loading huge tif file
HosseinMehr 9-Oct-07 6:30

Re: problem on loading huge tif file
Anthony Queen 24-Oct-07 20:21

Thanks a lot!
fahimb 26-Sep-07 14:50

C# version of image control [modified]
Gosta Forsum 3-Sep-07 23:42

Re: C# version of image control
JBoinker 18-Aug-11 12:35

Licensing?
Jeff Blankenburg 19-Jul-07 11:06

Re: Licensing?
Anthony Queen 19-Jul-07 12:10

I appreciate your diligence and honesty. Having openly posted the code here, I could never enforce a license. I do, however, ask that you put a reference to the article in your code as a comment. Perhaps something like this:

```
'******************************************************************************
'* This code is taken, with permission, from Queens_ImageControl for use
'* by
'*
'* The code for Queen's_ImageControl was written by Anthony Queen
'* and is available at http://www.theCodeProject.com
'* you are free to redistribute it and/or modify it as you see fit.
'*
'* Queens_ImageControl is distributed in the hope that it will be useful,
'* but WITHOUT ANY WARRANTY; without even the implied warranty of
'* MERCHANTABILITY or FITNESS FOR A PARTICULAR PURPOSE.
'*
'******************************************************************************
```

Please shoot me an email when you are done and let me know how it worked out for you. I'd be curious to know what you did.

Thanks,
Tony

p.s. Don't forget to vote.

It's only when you look at an ant through a magnifying glass on a sunny day that you realise how often they burst into flames.

Reply · Email · View Thread · Permalink · Bookmark

VERY wide or high images.... > 32767
larsake 15-Jul-07 12:41

Re: VERY wide or high images.... > 32767
Anthony Queen 16-Jul-07 11:11

Re: VERY wide or high images.... > 32767
Anthony Queen 16-Jul-07 11:39

Re: VERY wide or high images.... > 32767
larsake 16-Jul-07 13:45

Re: VERY wide or high images.... > 32767
Anthony Queen 19-Jul-07 13:24

Re: VERY wide or high images.... > 32767
Damir1 26-Jul-07 15:25

Re: VERY wide or high images.... > 32767
Anthony Queen 31-Jul-07 9:41

Reading Group 4 Fax Encoding compressed TIF
helpneeded_abcd 12-Jul-07 5:54

Refresh « Prev 1 2 3 **4** 5 6 7 8 9 10 Next »

General News Suggestion Question Bug Answer Joke Praise Rant Admin

Use Ctrl+Left/Right to switch messages, Ctrl+Up/Down to switch threads, Ctrl+Shift+Left/Right to switch pages.

Permalink | Advertise | Privacy | Terms of Use | Mobile
Web01 | 2.8.151126.1 | Last Updated 31 Oct 2009

Select Language

Layout: fixed | fluid

Article Copyright 2006 by Anthony Queen
Everything else Copyright © CodeProject, 1999-2015
