# Supplementary material for: Optimized criteria for locomotion-based healthspan evaluation in C. elegans using the WorMotel system
Source: PLoS One. 2020 Mar 3;15(3):e0229583. doi: 10.1371/journal.pone.0229583 (PMC7053758; doi:10.1371/journal.pone.0229583)
Supplement: S2 File — This includes relevant scripts (See Tutorial). (ZIP) [file pone.0229583.s020.zip › S Tutorial/WMdatanalysis_tutorial.pdf]

WORMOTEL DATA ANALYSIS:

TUTORIAL

Areta Jushaj[1], Matthew Churgin[2], Bowen Yao[2], Miguel De La Torre[2], Christopher Fang-Yen[2] and Liesbet Temmerman[1]

[1] Animal Physiology and Neurobiology, Department of Biology, KU Leuven, Leuven, Belgium

[2] Department of Bioengineering, University of Pennsylvania, Philadelphia, USA

For further questions, please contact areta.jushaj@kuleuven.be or liesbet.temmerman@kuleuven.be

Table of Contents

1. Introduction 2

2. Determination of pixel difference data from raw images 3

2.1. (Re)naming files 4

Context 4

Instructions 4

2.2. Differential image analysis 7

Context 7

Instructions 10

3. Extracting quantifiers of lifespan and health from pdata 26

3.1. Context 26

3.2. Convert pdata.mat to xls format 28

3.3. Calculate baseline and stimulated activity 29

3.4. Compile individual worm activity declines 30

3.5. Calculate life-and healthspan metrics 32

3.6. Working with life- and healthspan quantifiers 38

3.7. Calculate integrated activity and compare worm populations 38

4. Conclusions 40

1. Introduction

The goal of this tutorial is to guide new users through WorMotel data processing. Thereto, we collected a series of images of a WorMotel plate as described in Materials and Methods of Jushaj *et al.*, 2019. A test set for this tutorial is available upon request (approx. 30 GB).

The analysis consists of two major steps: determination of (1) pixel difference data from raw images and (2) life- and healthspan metrics from pixel difference data. In the image processing step, activity is extracted from raw image data by calculating the differences in pixels between images separated by a user-defined time interval (see Materials and Methods, Jushaj *et al.*,2019), this for each day and per individual well (thus, worm). Series of calculated pixel differences per well are then used in step (2) to calculate daily activity per worm per day, as explained in Jushaj *et al.*, 2019. This value is subsequently used to compile activity declines for individual worms over their lifespan. Based hereon, life- and healthspan quantifiers, i.e. lifespan (LS), healthspan (HS) and total days of health (TDH), are defined per well as described in Jushaj *et al.*, 2019. Derived metric such as health(span) ratio (H(S)R) are then calculated and can be compared at the population level, for which integrated activity (IA) is also calculated (Jushaj *et al.*, 2019). These values are also compared to internal control values to assist in candidate analysis.

To complete the analysis, active Matlab® and Microsoft Excel licenses are required, preferably versions released after 2017. The scripts that exert the desired functions and calculations have been stored in two directories, corresponding to the two major steps of the analysis. Basic understanding of programming is beneficial, but the tutorial aims to clarify the use of the analysis scripts to absolute beginners.

For this tutorial, data are used from a WorMotel plate of 240 wells that has been divided in 4 groups according to quadrant design (Figure 1). Each group contains 60 worms that were fed either RNAi bacteria containing the L4440 vector backbone (Figure 1 IC region), or the same vector containing the sequence of a gene of interest (Figure 1 Gene 1-3 regions).

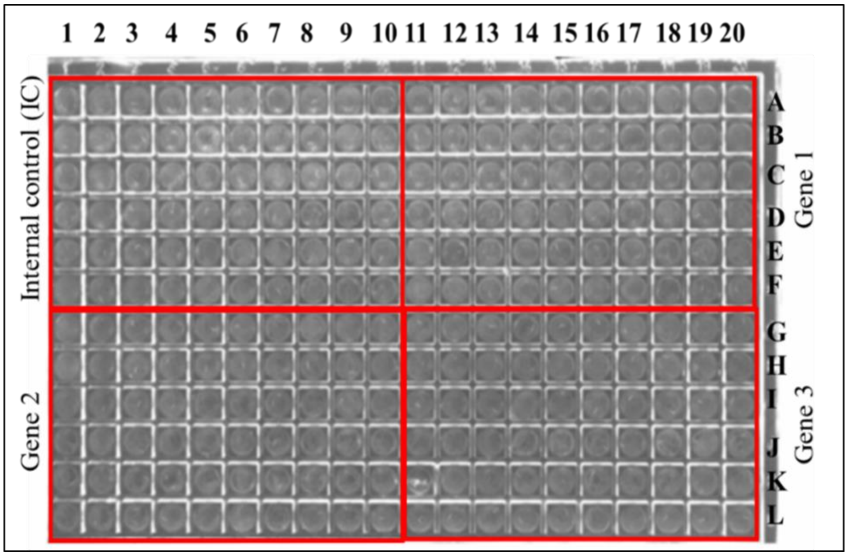

Figure 1

2. Determination of pixel difference data from raw images

All required Matlab® scripts for this step are available in the folder “Step1_Imageprocessing”. All scripts are needed for data analysis: even if the relevance of the script is not obvious, don’t delete or move any of the scripts. Be aware of the location (folder, path) where you chose to save the scripts, as the analysis requires this information.

2.1. (Re)naming files

Context

A WorMotel experiment will lead to a collection of image files, ideally in *.png* format (Figure 2). For analysis, it is required that the files have the name format 'YYYY-MM-DD (HH-MM-SS).png'. If your pictures were not stored with this name format, you will need to convert the files. The "***Convertdate_png.m***" script derives the required information from the time stamp that was created for the image when it was saved.

> **Note:** In some cases, it is possible that you collected 'empty' (0 kB) files. This typically happens when you programmed the system to collect more data than the remaining storage capacity of the drive. It is advised to delete these artifactual pictures upfront, as they will lead to errors in later steps.

The middle of every monitoring period should contain a white image due to pixel saturation caused by blue light stimulation (Figure 3). Following steps should be followed to rename the files to their appropriate time-stamps:

Instructions

Step 1. Open the script called "***Convertdate_png***.m" in Matlab® by double clicking.

Step 2. Set the Matlab® working directory to the location of the folders that need to be renamed. To do so, click the 'browse for folder' icon and select the location/path via the pop-up menu. The black arrow (Figure 4) indicates where in the script you can choose the format of your images.

Step 3. Click *run* (green play arrow in the 'Editor tab') and a pop-up menu will prompt you to select 'Add to Path'.

Step 4. Another pop-up window will prompt you to select the first image in the series that needs to be renamed. The command window will now indicate the progress (Figure 5), or deliver a message in case of error. After completion, the files will be renamed in the right format (Figure 6).

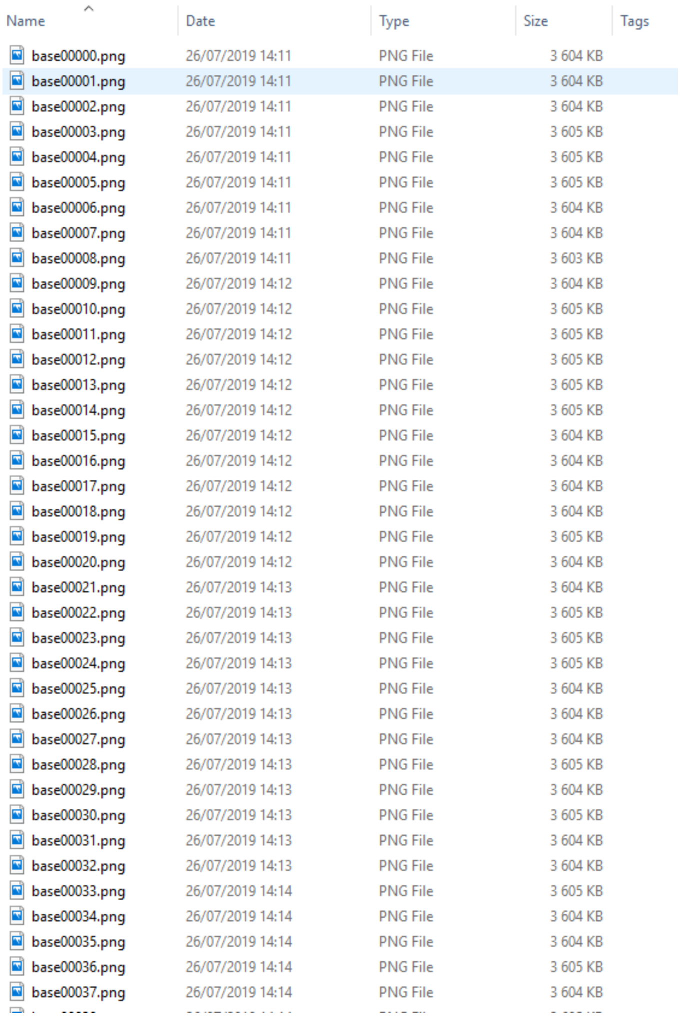

Figure 2

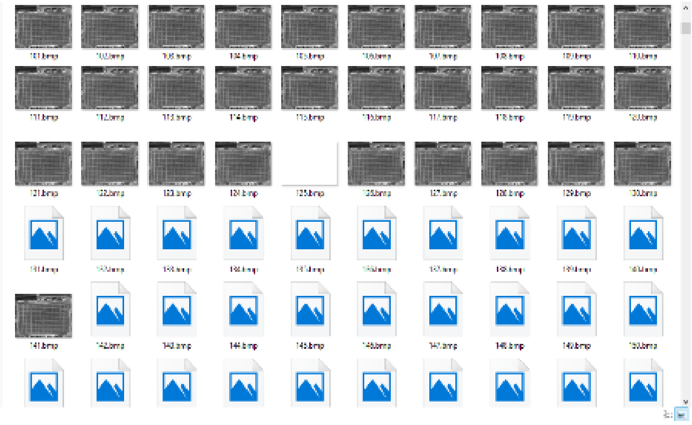

Figure 3

```
% convert files in a directory to format year-month-day (hour-min-sec)
% for example: 2016-05-28(14:14:32)

defaultpath = '/';

[FileName,PathName,FilterIndex] = uigetfile('*.png', 'Select 1 file',defaultpath);

defaultpath = PathName;

dirlist = dir([PathName '*.png']);

numfiles = length(dirlist);
for j=1:numfiles
    fname = dirlist(j).name
    [Y, M, D, H, MN, S] = datevec(dirlist(j).datenum);
    newfilename = [num2str(Y, '%04d') '-' num2str(M, '%02d') '-' num2str(D, '%02d') ' (' num2str(H, '%02d')...
        '-' num2str(MN, '%02d') '-' num2str(S, '%02d') ').png'];
    movefile([PathName fname], [PathName newfilename]);
end

```

Figure 4

```
Command Window
fname =

    'base00246.png'


fname =

    'base00247.png'


fname =

    'base00248.png'
```

Figure 5

2017-07-15 (04-18-24).png	15/07/2017 4:18	PNG File	14 763 KB
2017-07-15 (04-18-30).png	15/07/2017 4:18	PNG File	14 763 KB
2017-07-15 (04-18-35).png	15/07/2017 4:18	PNG File	14 763 KB
2017-07-15 (04-18-41).png	15/07/2017 4:18	PNG File	14 763 KB
2017-07-15 (04-18-46).png	15/07/2017 4:18	PNG File	14 763 KB
2017-07-15 (04-18-52).png	15/07/2017 4:18	PNG File	14 763 KB
2017-07-15 (04-18-57).png	15/07/2017 4:18	PNG File	14 763 KB
2017-07-15 (04-19-02).png	15/07/2017 4:19	PNG File	14 763 KB
2017-07-15 (04-19-08).png	15/07/2017 4:19	PNG File	14 763 KB

Figure 6

2.2. Differential image analysis

Context

The differential image analysis step calculates pixel differences between consecutive images at a set interval (see Materials and Methods, Jushaj *et al.*,2019) for each monitoring time. The script is embedded in a graphic user interface (GUI). Some steps of the analysis are hidden behind the GUI's commands, which is why we here provide some context.

Following folders are important for this analysis:

- The "image folder": containing the to-be-processed images. This folder needs to have a five-character alphanumerical name, such as EXP01.
- The "analysis" folder: an automatically generated parent folder containing following subfolders
    - The "results folder": automatically generated folder onto which all calculated pixeldifference data is saved. This folder is automatically given the same name as the "image folder", thus *e.g.* EXP01.

- “ROItemplate” folder: onto which data important for Region Of Interest (ROI) definition is copied.

During the tutorial, these folders are referred several times. Before the analysis starts, you should be aware of the location/path of the used script, of the “image folder” and of the “analysis” folder.

> **Note:** To differentiate folders with predefined names (*e.g.* the "analysis" folder) from folders that can be named according to the user's preference (*e.g.* "image folder"), different positions of quotation marks are used throughout this tutorial.

> **Note:** The “analysis” folder can contain multiple results folders for different analyzed experiments.

> **Note:** The “image folder” and “results folder” will have exactly the same name for the same WorMotel plate and differ therefore only in location (and content). Caution is advised as the content of these folders is completely different.

Overall, the differential image analysis consists of three major parts: (1) initiation and definition of ROIs, (2) assignment of the path of the “analysis” folder (to be generated there) and (3) definition of the location of the “image folder”. To initiate the script in step (1), you are required to select the path in which the used script is located. This step is necessary for the GUI to access the correct subscripts during the analysis. Next, separate WorMotel wells on the images need to be identified. For this, ROIs are manually defined, confining one well to one ROI. The script employs the first image in the series to define the ROIs and then conveys the same ROIs to all images based on the pixel-location of the ROI boundaries of the first image. This is the reason

why the location/path of the "image folder" is requested twice: once to find the first image, and once more in part (3).

Definition of the ROIs results in the generation of additional files: (a) 'inits.mat' file and (b) ROI files. The 'inits.mat' file contains the information regarding the pixels in the first image and the ROIs. There are two ROI files: an overlay image and a *.mat* file containing the coordinates of the ROIs. These files are initially generated in the "image folder" but are then automatically copied into the "ROItemplate" folder (located in the "analysis" folder). This action is the reason why the location of the "analysis" folder will be asked twice during the differential image analysis (see Instructions). Here, but also later: the location/path of the "image folder" is required again so that the calculation of the pixel differences can start. As the script progresses, it creates and uses all above-mentioned files to create the **pixel difference data files (pdata files)**. At the end of the run, following folders and files are added to the "results folder".

- "pdata *X* sec" folder: containing all the calculated pixel differences per day for an *X* sec interval between analyzed images

- "pdata 540 sec" folder: containing the calculated pixel differences per day for a 540 sec interval between analyzed images

- 'imagetimearray.mat' file: containing the time for each monitoring period (begin and end)

Instructions

Preparative steps

A first goal of the overall workflow is the calculation of activity represented by pixel differences (saved in the pdata files) observed in the images for each worm, each day. For this, open the script '***StimulationImageAnalysisAlpha.m***' (SIA). It relies on different underlying scripts, also provided in the "Step1_Imageprocessing" analysis folder from this tutorial and run step-by-step to yield pdata files. Before initializing the script, we will go over settings that are adjustable to your preferences.

- ✓ Line 204 (Figure 7) states the number of wells per plate. As different formats of the WorMotel exist (*e.g.* 48-well plates), this value is important.
- ✓ Lines 232-235 (Figure 8) allocate wells to experimental conditions. Data for this tutorial belong to four experimental groups (Figure 1). Wells are numbered according to row formatting, *i.e.* rows contain wells 1 to 20 (row 1), 21-40 (row 2), etc. Therefore, the internal control group of this tutorial is located in wells 1:10 21:30 41:50 61:70 81:90 101:110. It is important to assign wells to appropriate groups here, prior to running the script, as this cannot be done afterwards.
- ✓ Lines 264-269 (Figure 9) show the set time interval (in seconds) between images for calculation of lifespan and activity (healthspan). Should you wish to (re)analyze data for a different interval, this setting needs to be changed and the analysis needs to be (re)run in order to create appropriate pdata files. Based on Jushaj *et al.*, 2019, this value is currently set to 100 s.

- ✓ Lines 307-308 (Figure 10) permit to modify the accepted monitoring time. For our standard analysis (20 min monitoring period per day), monitoring periods shorter than 18 or longer than 32 mins are discarded.

```
% Well count set to 240
% Change here if necessary!
wellCount=240;



```

✓

Figure 7

```
% load well info
if exist([destAnalysisFolder filesep 'Well info.mat'])
    load([destAnalysisFolder filesep 'Well info.mat']);
elseif exist([destAnalysisFolder filesep 'Well info.txt'])
    getGenotypeInformation(wellCount, destAnalysisFolder);
    load([destAnalysisFolder filesep 'Well info.mat']);
else
    genotypes{1} = [1:10 21:30 41:50 61:70 81:90 101:110];
    genotypes{2} = [11:20 31:40 51:60 71:80 91:100 111:120];
    genotypes{3} = [121:130 141:150 161:170 181:190 201:210 221:230];
    genotypes{4} = [131:140 151:160 171:180 191:200 211:220 231:240];
end
handles.genotypes=genotypes;
handles.genono=size(genotypes,1);

```

Figure 8

```
srcImageFolder=handles.srcImageFolder;
rootFolder=uigetdir(srcImageFolder,'Select where analysis scripts exist');
cd(rootFolder);
addpath(genpath(rootFolder));   % add all subfolders to matlab path
templateROIid=handles.templateROIid;
maxActivity = 400;       % the highest value of activity shown in heatmap
analysisIntervalForLifespan = 60*9;     % 10 min
analysisIntervalForActivity = 100;      % 20 sec
handles.maxActivity = maxActivity;
handles.analysisIntervalForLifespan = analysisIntervalForLifespan;
handles.analysisIntervalForActivity = analysisIntervalForActivity;

```

Figure 9

```
maxTimeIntervalSec = 60; % this two values are hard coded, change when necessary
minDurationMin = 18;
activePeriodsArray = SearchActiveImagingPeriods(imageTimeArray, maxTimeIntervalSec, minDurationMin);
disp('Done');
```

Figure 10

Analysis initiation and ROI definition

Step 1. After potential adjustments in the script, click '*Run*' to load the GUI. On the left side of the GUI, you will see pre-commands: information that needs to be completed before starting the analysis. The right side of the GUI will not be discussed in this basic tutorial.

Step 2. To start the analysis, click the '*Initialize and define ROIs*' button (Figure 11). Required commands are always shown at the top of the pop-up window.

Step 3. In the initial pop-up, select the folder in which the active SIA script is located (Figure 12).

Step 4a. Click the first picture of your experiment (located in the "image folder", Figure 13), whereupon the script requires input regarding the format of the WorMotel plate to be analyzed (Figure 14).

Step 4b. In this example, fill out "3" as data were collected for a 240 well plate. Leave other values unchanged and click '*ok*'.

Step 4c. You should now see the first picture of your experiment, which will allow you to indicate the regions of interest (Figure 15-21). Careful selection is important, as indicated ROIs will be maintained throughout analysis and sloppy selection will influence results – for example, ROIs missing worms on a side of a well may call the animal as dead, even when in fact alive but tangential to the ROI. Similarly, missing part of the worms will lead to inaccuracy of the measured activity. That being said, perfect ROIs are hard to achieve, and optimal selection takes some practice. Note that ROIs are sensitive to mistakes in the shape of the plate (or mold used to make the plate): uneven molds lead to distorted pictures, therefore inaccurate assignment of ROIs. High-quality data analysis begins with a high-quality experiment!

Step 4d. To define the ROIs, click the middle of the left upper well (well 1), followed by the middle of the right upper well (well 20), the left bottom well (well 221) and the right bottom well (well 240). This information is used to calculate the middle of each well in the plate. You can evaluate ROI selection by visually assessing how well the calculated middle corresponds to the actual middle of the wells.

Step 4e. After this, choose a well of your liking that is preferably close to the middle of the plate and whose middle has been chosen well. Click the left upper corner of this well (Figure 19-21) followed by the right bottom corner to establish the borders of the well. Don't be too strict, as

you don’t want to miss parts of wells, but also do not be too generous, as overlaps between different wells can occur that might skew results afterwards (*e.g.* calculated activity cannot be due to a neighboring worm).

> **<u>Note:</u>** It is possible that during an experiment, plates move slightly within the field of view due to robotic or manual handling. Because corresponding shifts in ROIs can cause huge inaccuracies for the automated calculation of life- and healthspan values, small but uniform ROI changes between images are accounted for automatically.

Step 5. Now click outside the picture area and the script will ask to select the folder containing the ‘inits.mat’ file, created upon defining the ROIs, *i.e.* the “image folder”.

Step 6. You will need to select a location/path to generate the “analysis” folder (Figure 22). This results in the immediate co-generation of its subfolders, *i.e.* the “results folder” and the “ROItemplate” folder, as described above. You are again asked to select the first image of the series; after which you will need to name the interventions you are studying (Figure 23). At the end of this step, the script pauses, indicating that the initial part ‘Initialize and define ROIs’ has ended (Figure 24).

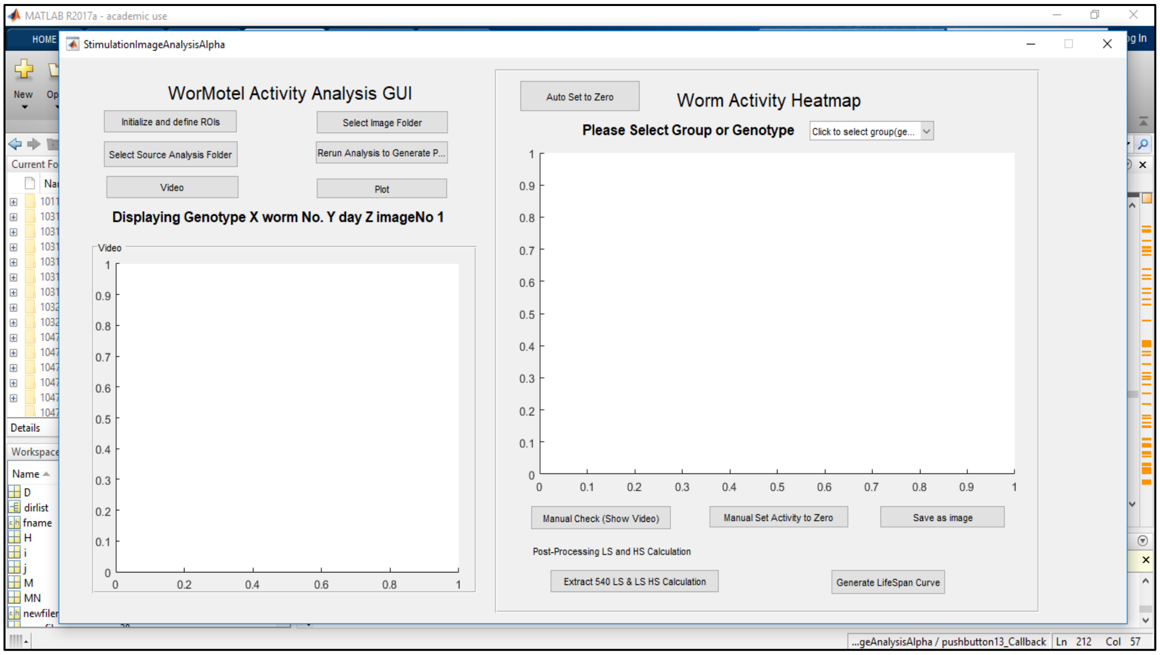

Figure 11

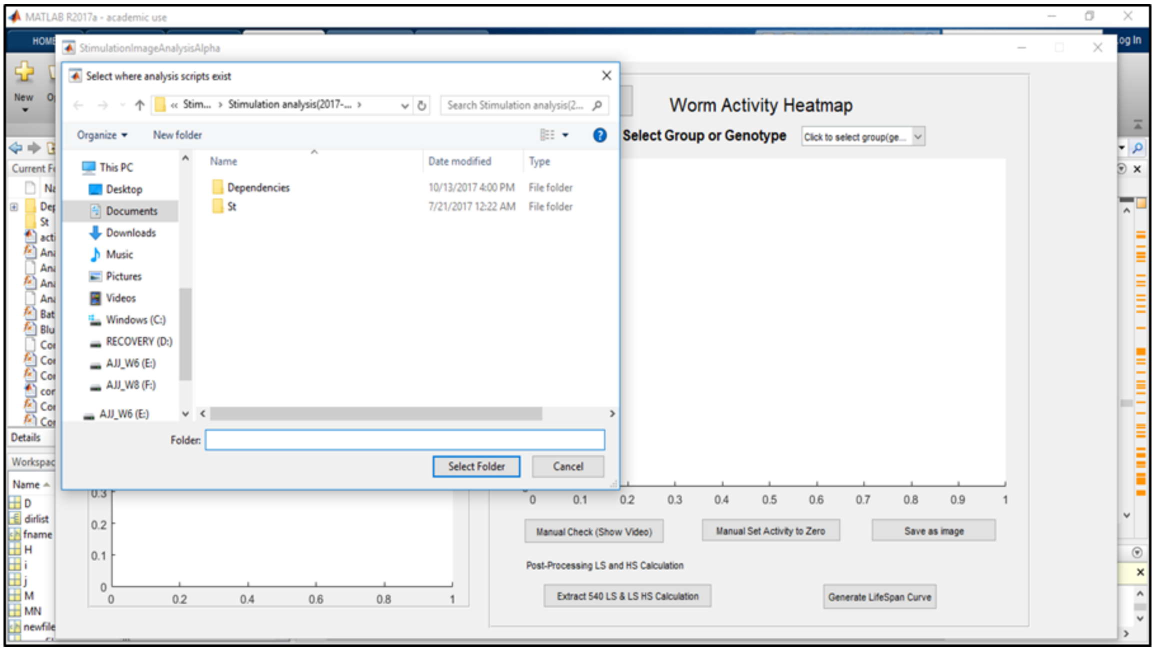

Figure 12

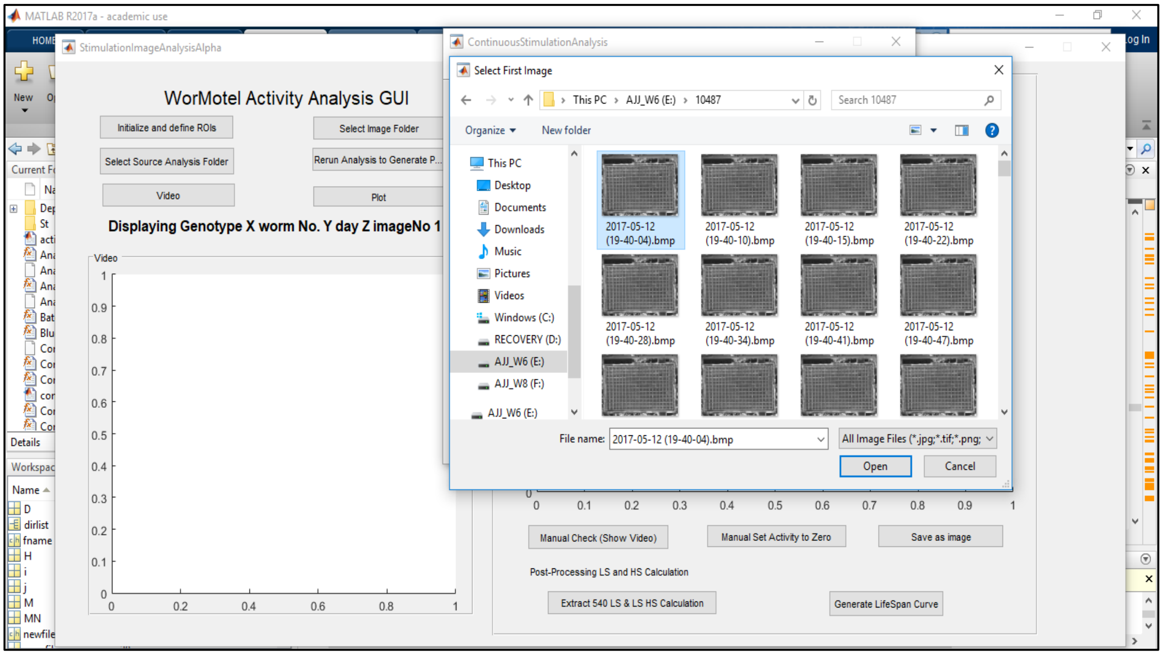

Figure 13

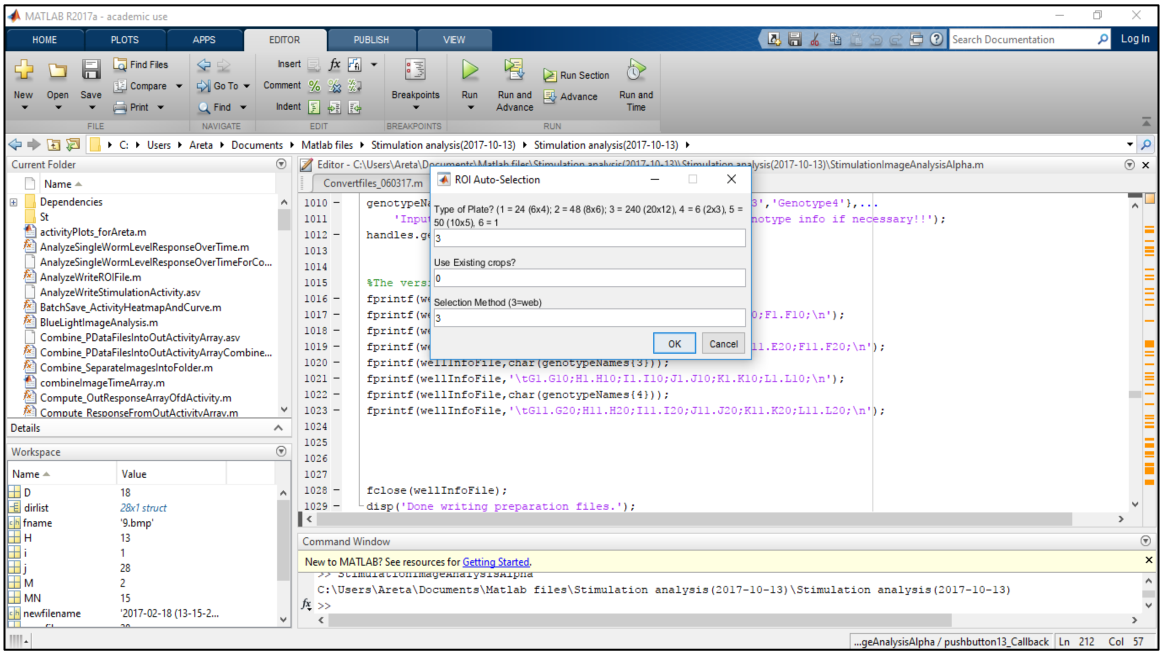

Figure 14

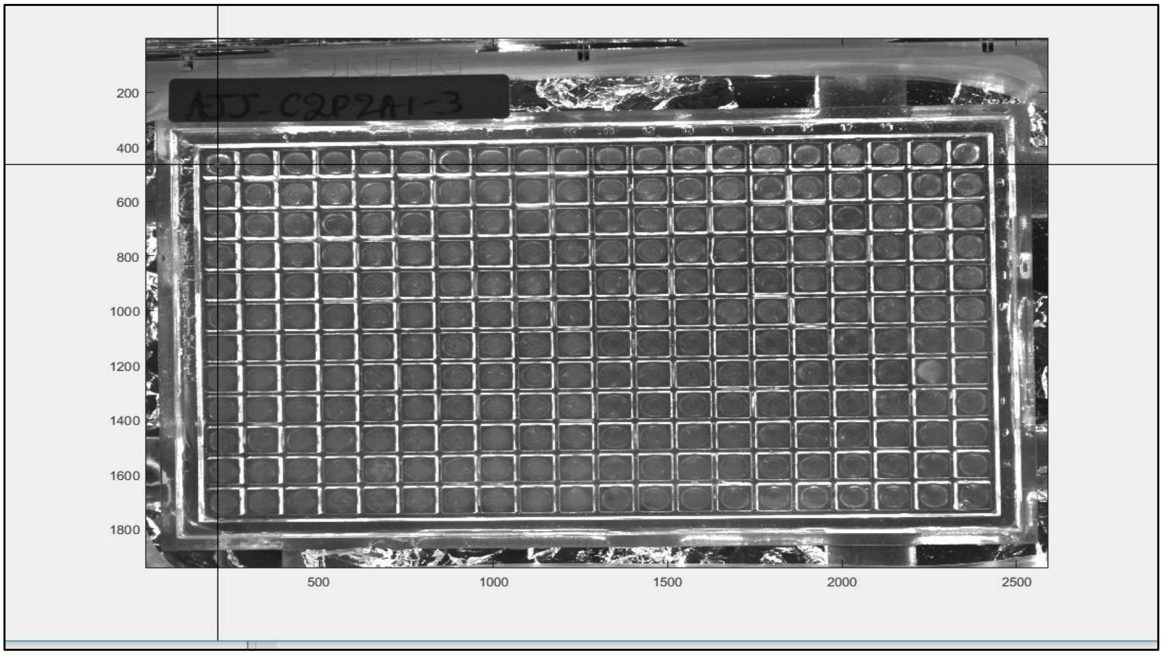

Figure 15

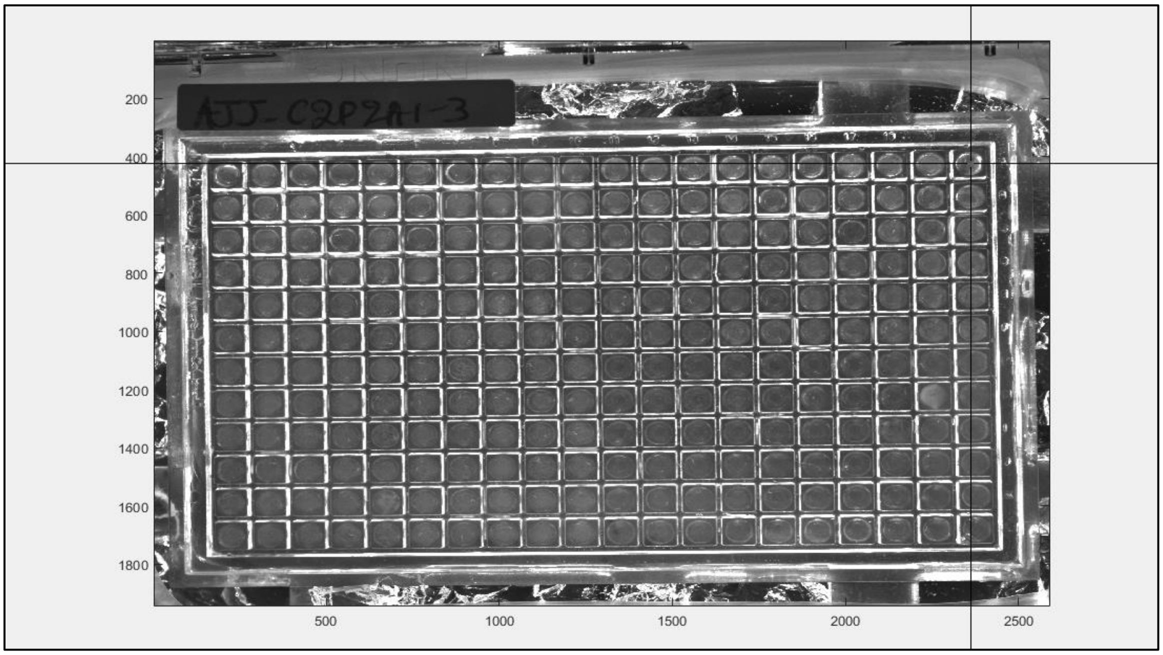

Figure 16

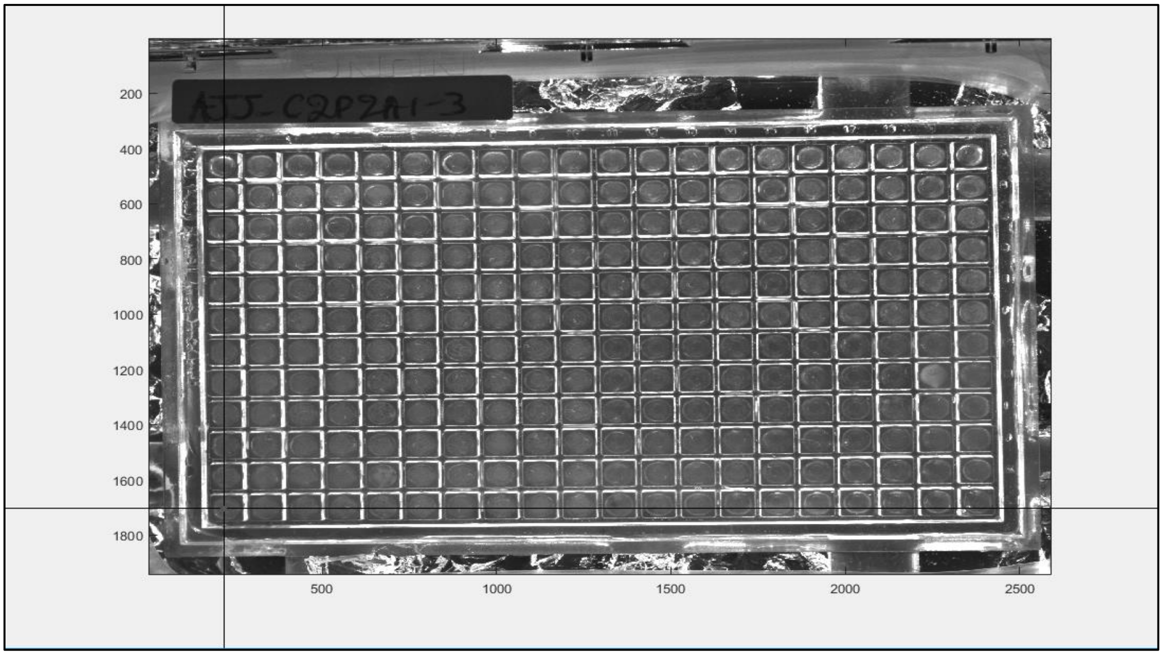

Figure 17

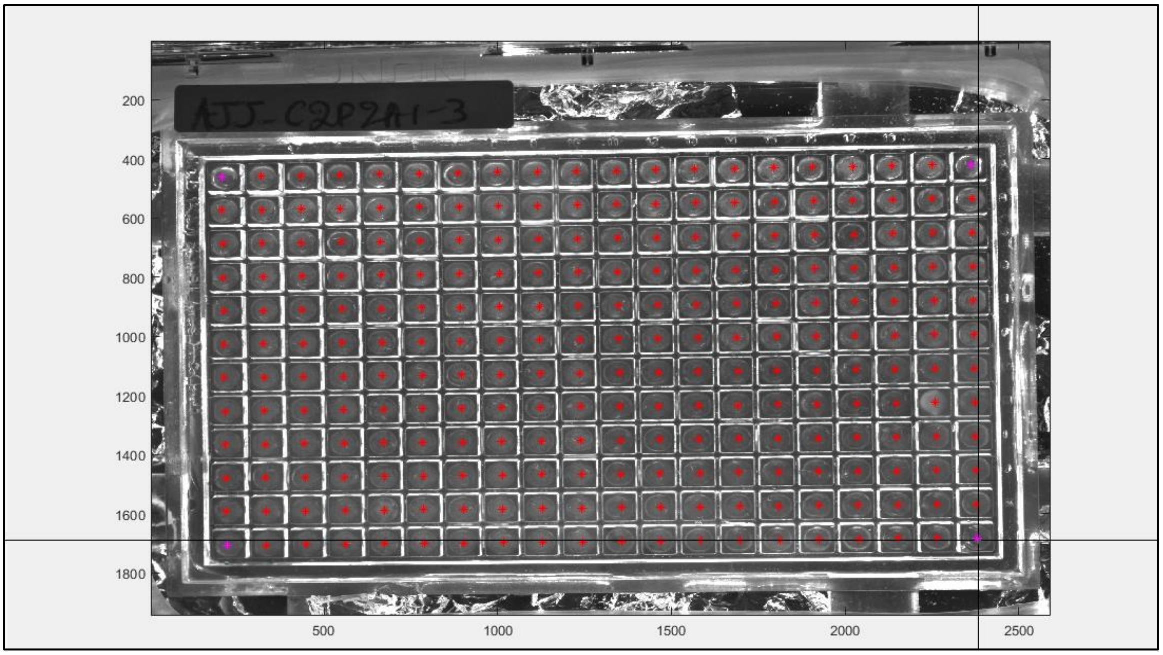

Figure 18

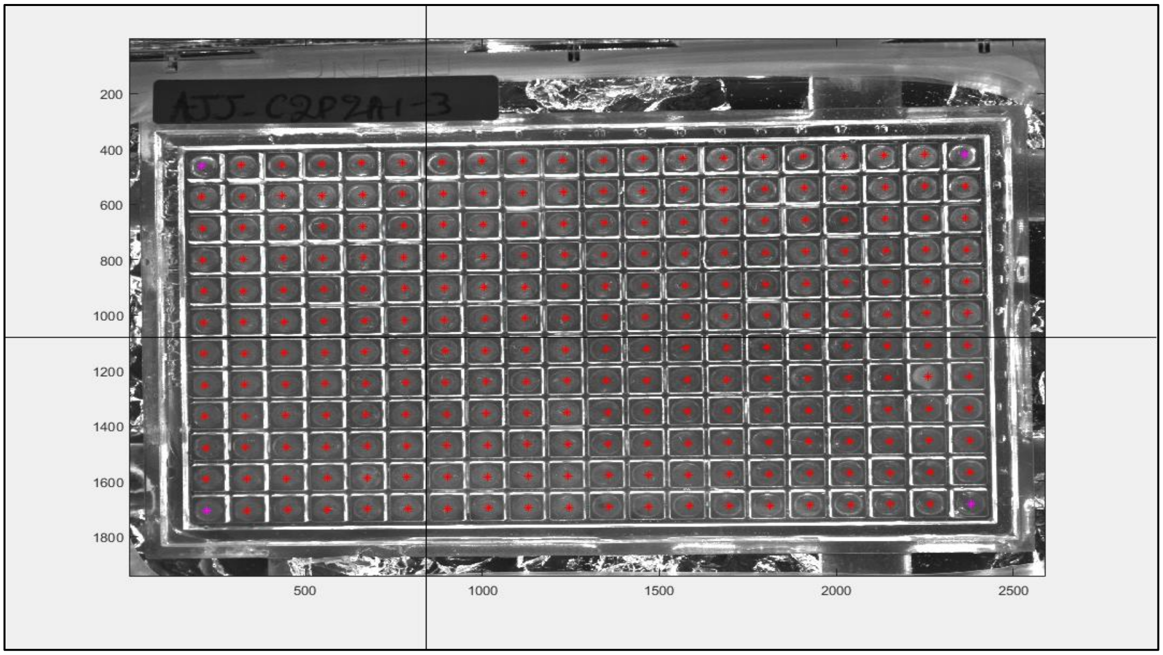

Figure 19

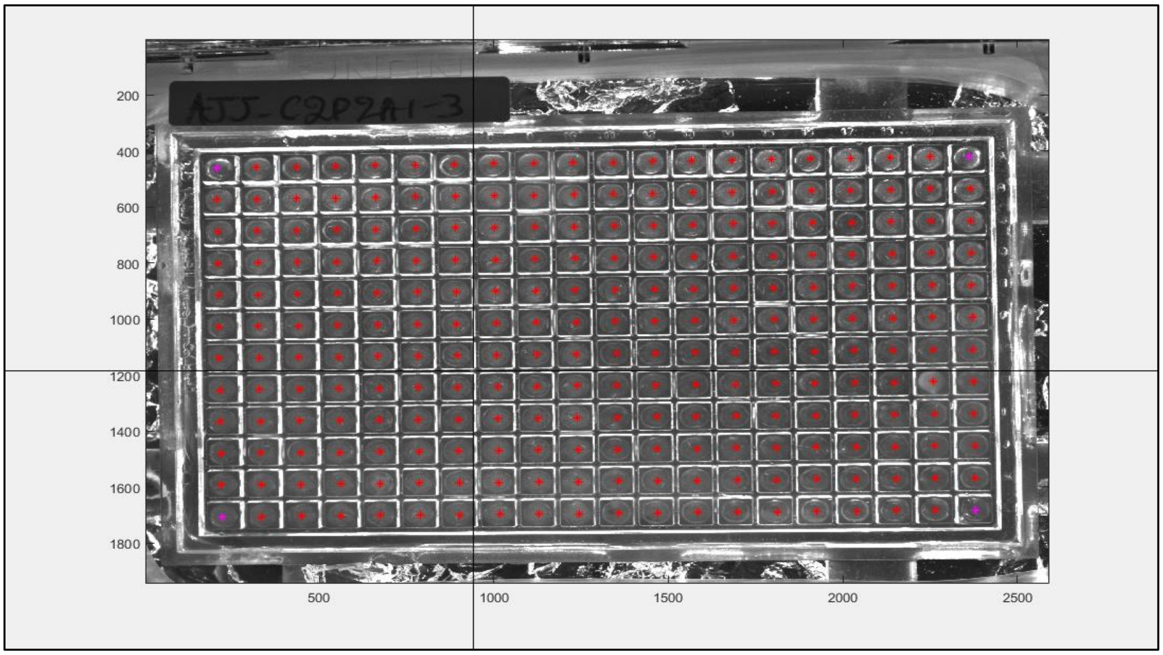

Figure 20

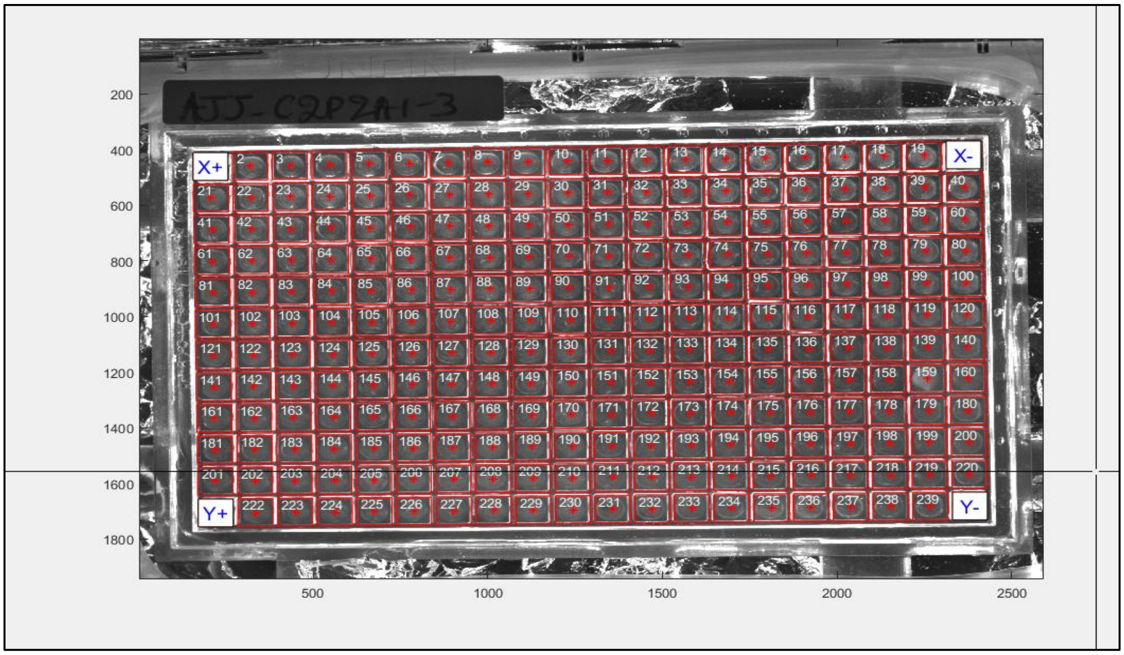

Figure 21

"Analysis" folder selection

Step 7. To continue the paused SIA script, click '*Select Source analysis folder'* (Figure 11). You are prompt to select the "results folder", with the same name as the "image folder" that is being analyzed. This folder now also contains automatically generated files containing well information and experiment start time.

"image folder" selection

Step 8. Click "*Select Image Folder*" to select the "image folder".

Analysis run and generated data

Step 9. Click '*Rerun Analysis*' to start the calculation of pixel differences and generation of pdata files. Select once more the directory in which the SIA script is located. The script is now running, and analysis time will depend on the amount of days that you have monitored as well as the processing power of your computer. For example, analyzing 40 days of imaging data takes

about 1 hour on an i7 1.8GHz with 12G RAM and 64bit Windows OS. This step creates pdata files, a “pdata (interval X sec)” folder, a “pdata (interval 540 sec)” folder, and an ‘Imagetimearray.mat’ file (Figure 25) and completes a functional unit in data processing. For this tutorial and based on Jushaj *et al.*, 2019, 100 s intervals were chosen by default (see ‘preparative steps’ - Lines 264-269 and Figure 9).

Within the “pdata (interval X sec)” folder [here: “pdata (interval 100 sec)”], pdata files per monitoring period have now been created (Figure 26). These pdata files are named based on the number of images in the EXP01 folder, *e.g.* “pdata2.mat” for the first day of the monitoring period and “pdata540.mat” for day 20 of the monitoring period of this tutorial. Exact numbers may differ for the analysis of other data, but the order of imaging periods will always correlate with the order of file numbering.

If it is desired to open a certain pdata file, double click the file, followed by clicking “pdata” in the import wizard (red arrow in Figure 27). You can preview the raw data on the right side of the import wizard window. Wells contain calculated pixel differences for the set interval (here: 100 s), Columns list individual wells (=worms), each row contains a pixel difference values for a single comparison of 2 images of the daily imaging period. Blue light stimulation is recognized by consecutive “-2” values (Figure 28).

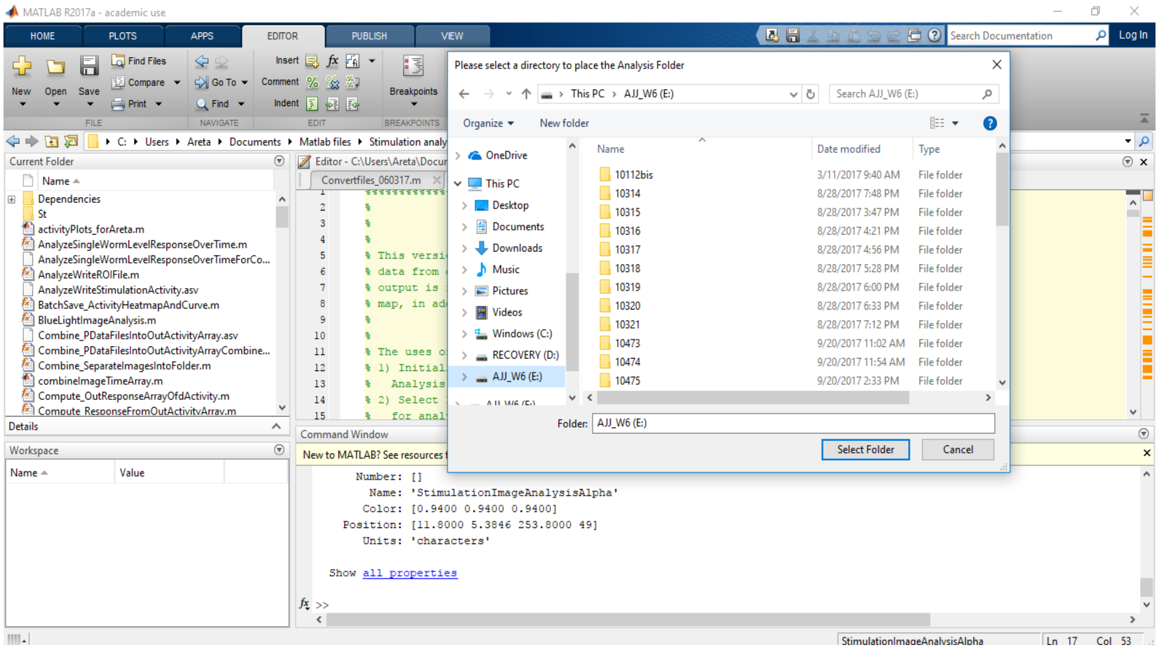

Figure 22

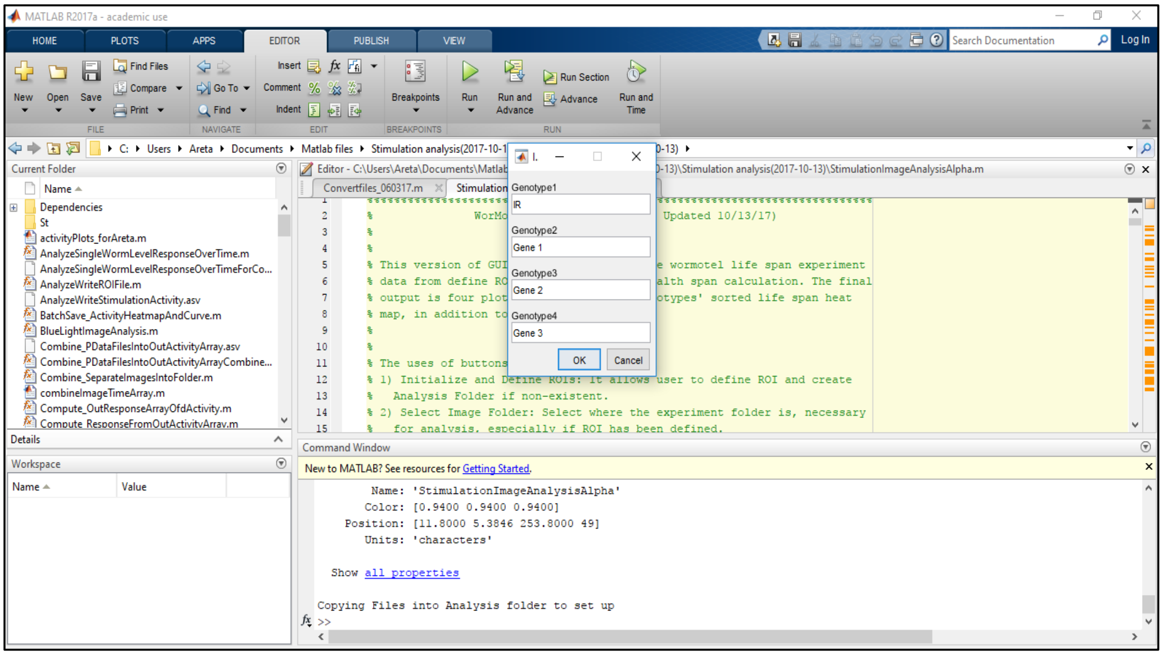

Figure 23

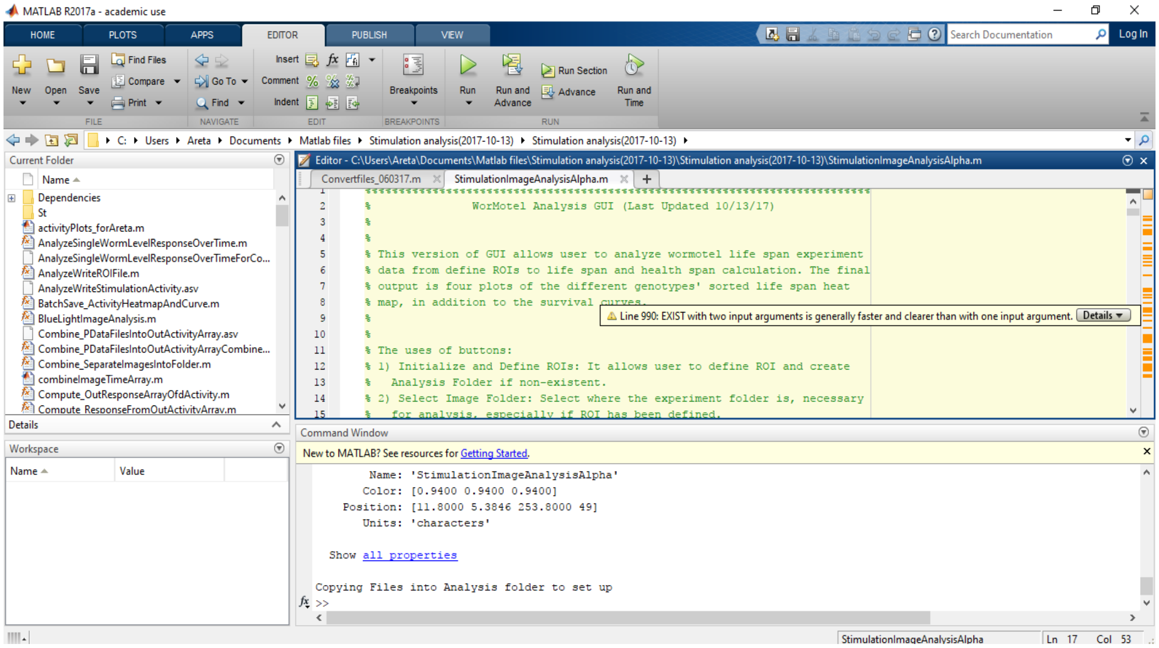

Figure 24

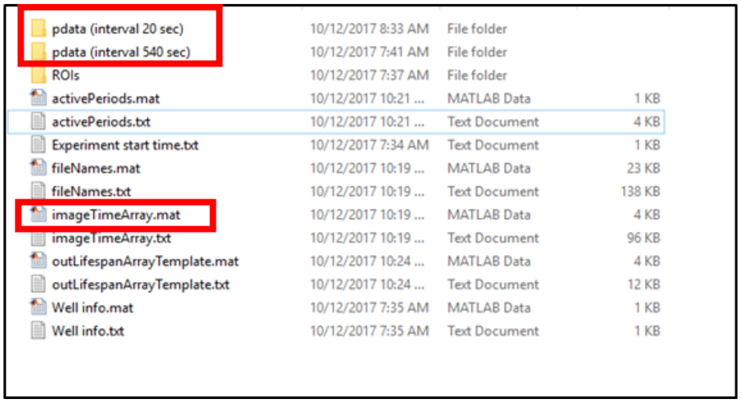

Figure 25

Name	Date modified	Type	Size
pdata2.mat	10/12/2017 10:26 ...	MATLAB Data	40 KB
pdata198.mat	10/12/2017 10:28 ...	MATLAB Data	36 KB
pdata404.mat	10/12/2017 10:30 ...	MATLAB Data	50 KB
pdata610.mat	10/12/2017 10:32 ...	MATLAB Data	40 KB
pdata817.mat	10/12/2017 10:33 ...	MATLAB Data	21 KB
pdata1022.mat	10/12/2017 10:35 ...	MATLAB Data	17 KB
pdata1230.mat	10/12/2017 10:37 ...	MATLAB Data	27 KB
pdata1437.mat	10/12/2017 10:39 ...	MATLAB Data	7 KB
pdata1641.mat	10/12/2017 10:41 ...	MATLAB Data	5 KB
pdata1841.mat	10/12/2017 10:43 ...	MATLAB Data	3 KB
pdata2040.mat	10/12/2017 10:45 ...	MATLAB Data	3 KB
pdata2247.mat	10/12/2017 10:47 ...	MATLAB Data	2 KB
pdata2463.mat	10/12/2017 10:49 ...	MATLAB Data	2 KB
pdata2672.mat	10/12/2017 10:50 ...	MATLAB Data	2 KB
pdata2880.mat	10/12/2017 10:53 ...	MATLAB Data	2 KB
pdata3093.mat	10/12/2017 10:55 ...	MATLAB Data	2 KB
pdata3307.mat	10/12/2017 10:56 ...	MATLAB Data	2 KB
pdata3517.mat	10/12/2017 10:58 ...	MATLAB Data	2 KB
pdata3729.mat	10/12/2017 11:00 ...	MATLAB Data	2 KB
pdata3941.mat	10/12/2017 11:02 ...	MATLAB Data	2 KB
pdata4155.mat	10/12/2017 11:04 ...	MATLAB Data	2 KB
pdata4365.mat	10/12/2017 11:06 ...	MATLAB Data	2 KB
pdata4574.mat	10/12/2017 11:08 ...	MATLAB Data	2 KB
pdata4788.mat	10/12/2017 11:10 ...	MATLAB Data	2 KB
pdata5002.mat	10/12/2017 11:12 ...	MATLAB Data	2 KB
pdata5212.mat	10/12/2017 11:14 ...	MATLAB Data	2 KB

Figure 26

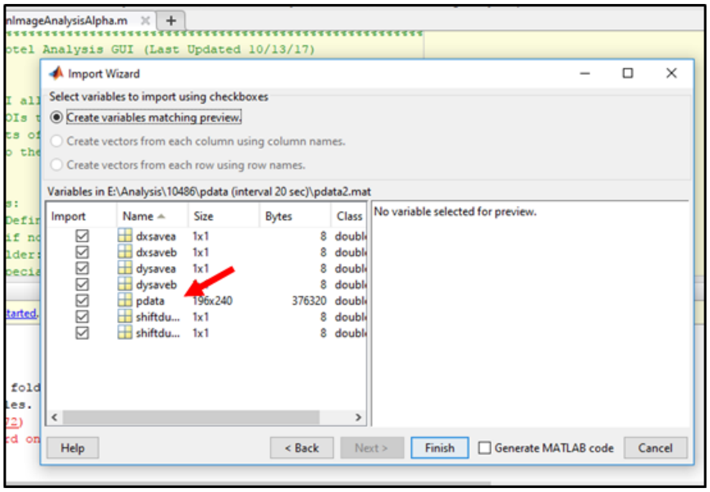

Figure 27

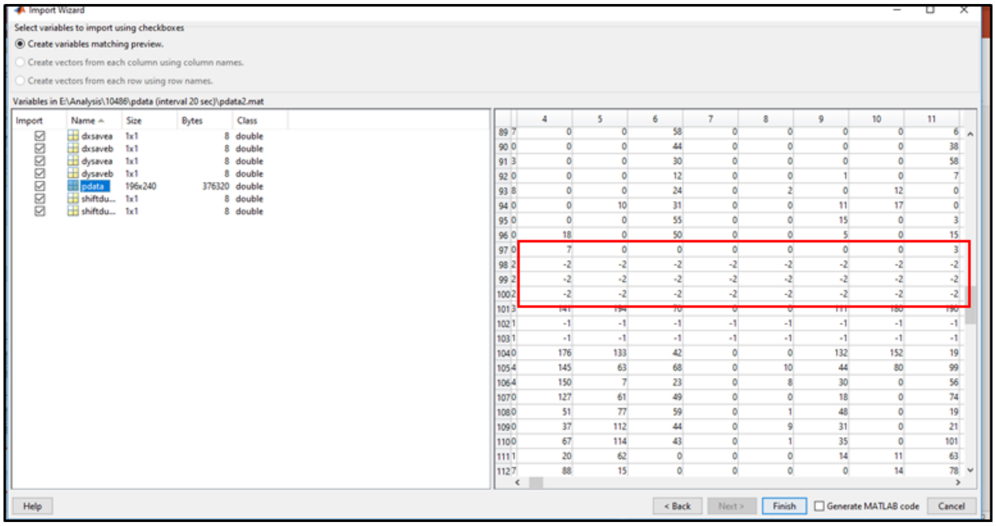

Figure 28

3. Extracting quantifiers of lifespan and health from pdata

3.1. Context

At the end of “2. Determination of pixel differences from raw images”, you will end up with a series of pdata files (Figure 26). Further analysis requires a distinction between pre- and post-stimulation data, *i.e.* data collected prior to, or after the blue light stimulus of the monitoring period. Here, only post-stimulation data are used, but the same principles apply to pre-stimulation data handling.

Via the instructions below, you will isolate post-stimulation data for each worm and summarize daily activity values (cf. pdata files) into one representative value per day (see Materials and Methods, Jushaj *et al.*, 2019). Newly created files with a series of single daily activity values for the worms will then be used for determination of lifespan and health quantifiers: lifespan (LS), total days of health (TDH), healthspan (HS), health(span) ratio (H(S)R), and integrated activity (IA) (see Jushaj *et al.*, 2019). Because the final aim is to compare the effect of (genetic or other) interventions on distinct worm populations in the WorMotel plate, distributions of individual lifespan and health parameters are then studied according to quadrant design (see Introduction).

Once assigned to groups, the individual quantifiers are copied into new files and adjusted for missing worms. Population activity declines are also copied into new files to calculate the ‘integrated activity’ (see Materials and Methods, Jushaj *et al.*, 2019).

In the last step, population averages for the measured and grouped quantifiers are calculated and compared to those of the internal control to create normalized quantifiers. Normalized population averages allow interpretation of results across multiple WorMotel plates, as it corrects for inter-plate variation.

By the end of the analysis, you will have obtained normalized values for LS, HR and IA (see Jushaj *et al.*,2019), here referred to as the three ‘coordinates’ of the population. These coordinates allow interpretation of relative changes in LS, HR and IA for the studied population in comparison to those of the control population on the same plate, and are particularly practical for the assessment of medium to high-throughput data. In the test set used in this tutorial, the interventions marked here Gene 1, Gene 2 and Gene 3 result in the following coordinates: **1.70**:**0.69**:**0.78** for Gene 1; **0.80**:**1.08**:**0.63** for Gene 2 and **0.97**:**0.90**:**0.63** for Gene 3. This means that for a worm population treated with *e.g.* RNAi targetting Gene 2, the average LS and IA are decreased by respectively 19% and 38%, whereas the average HR of the population is increased by 6% respective to internal controls. Please note that not all animals belonging to the Gene 2 group were dead by the end of this experiment (Figure 36). For this population, average LS and HR are not incomplete and thus result in inaccurate population coordinates.

Note: An internal control population is always characterized by coordinates 1:1:1.

To facilitate analytical progress, following folders are required as subfolders of a manually created parent folder to be named ‘post-processing results’:

- “excel pdata folder”: manually generated and containing the pdata files in *.mat* format (this folder can be renamed according to your preference)
- “metrics folder”: a manually generated folder to facilitate the calculation of lifespan and health quantifiers (this folder can be renamed according to your preference). It contains the following subfolders, always named exactly as follows:
    - “GTfiles”: manually generated folder, which is initially empty but will be used in step 3.5 to save lifespan and health quantifiers

   - “Plates”: a manually generated folder into which compiled activity decline files will be manually copied between steps 3.4 and 3.5.
   - “Timefiles”: a manually generated folder containing the time vector (see step 3.5)
- “LSHSdata”: manually created folder with this exact name
- “Activities”: manually created folder with this exact name
- “normalized coordinates”: manually created folder onto which the normalized LS, HR and IA will be saved (this folder can be renamed according to your preference).

Note: Use underscore instead of spaces in names of folders where the exact name was not specified by this tutorial, *i.e.* the “excel pdata”, “parameters” and “normalized coordinates” folders. For “GTfiles”, ‘Plates’, “Timefiles”, “LSHSdata” and “Activities” folders the names should exactly be as written in this tutorial.

All relevant scripts for the following instructions are located in the “Step2_Metrics” folder, where they are ordered according to the workflow thanks to an alphabetical prefix. VBA (Microsoft Excel) scripts act in modules, these are separate subscripts to exert different functions. It is customary to rely on a ‘calling module’, a parent module from which all other modules are activated to exert their function. This bypasses the need to activate every module individually and offers more streamlined scripts. To run a script, placing the cursor in the ‘calling module’ (thus making it the active window) and clicking ‘*run*’ is required. ‘Calling modules’ will be specified for each step in the following instructions.

3.2. Convert pdata.mat to xls format

Because you will go back and forth between Matlab® and VBA (Microsoft Excel) scripts, pdata files first need to be converted from *.mat* to *.xls* formatted files. It is prudent to copy the pdata

files of the desired time interval into a separate folder, *i.e.* the “excel pdata folder” and not use the original pdata files generated in the image processing step. To convert the files from *.mat* to *.xls*, use the “***A_convertPdataToExcel.m***” file in the “Step2_Metrics” folder. For this script, the Matlab® working directory needs to be the “excel pdata folder”.

3.3. Calculate baseline and stimulated activity

In order to split pre- from post-stimulation data, use the “***B_Splitstimulation.xlsm***” script, located in the “Step2_Metrics” folder. This script splits pdata files (in .xls format) by recognizing the “-2” values as the presence of an oversaturated white image. It also removes all “-1” values, caused by the absence of a pixel difference data point for that moment in the analysis (always true for the first rows). Lastly, the script summarizes the pixel difference data for each monitoring period in different activity metrics per worm (see Jushaj *et al.*,2019). Complete the following steps:

Step 1. Open the VBA script (“***B_Splitstimulation.xlsm***”) and click the ‘*Developer*’ tab. In this tab, click ‘*View Code’* (Figure 29). This will open an extra window, where the modules can be accessed for potentially required changes.

Step 2. In this script, the ‘calling module’ is conveniently called “Calling” (Figure 30). Double click this module to change the objFolder (Figure 31, black arrow) to the folder containing your XLS-formatted pdata files. It is advised not to have any other XLS files in this folder.

Step 3. Run the script by either clicking the “Results” button or by running the “Calling” module (Figure 30) as explained above.

This will add two additional sheets to the XLS pdata files, named Baseline and Stimulated.

3.4. Compile individual worm activity declines

For each worm, several metrics that describe its daily activity are calculated in section 3.3. As our goal is to evaluate activity decline for each worm, longitudinally over aging, these values are best summarized together in one file. For this, use "***C_Summarizeactivity.m***" in the "Step2_Metrics" folder (Figure 32). As indicated in the script, several adaptations regarding location of your data and pre- or post-stimulation data extraction can be made. The output of this script will be saved in the working directory of Matlab®, preferably the "excel pdata folder". In line 30 of the script, you can indicate the name of the resulting file, which should contain only 5 characters (*e.g.* EXP01), as required for further processing. By default, peak activity (see Materials and Methods of Jushaj *et al.*, 2019) is used to define daily activity of individual worms.

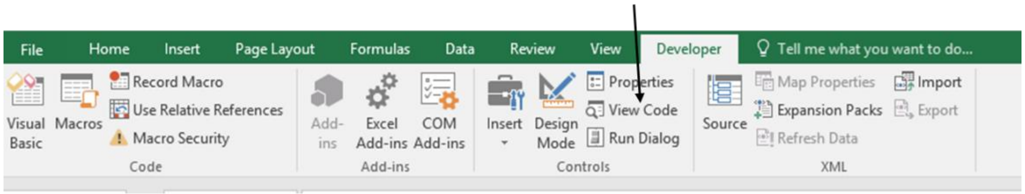

Figure 29

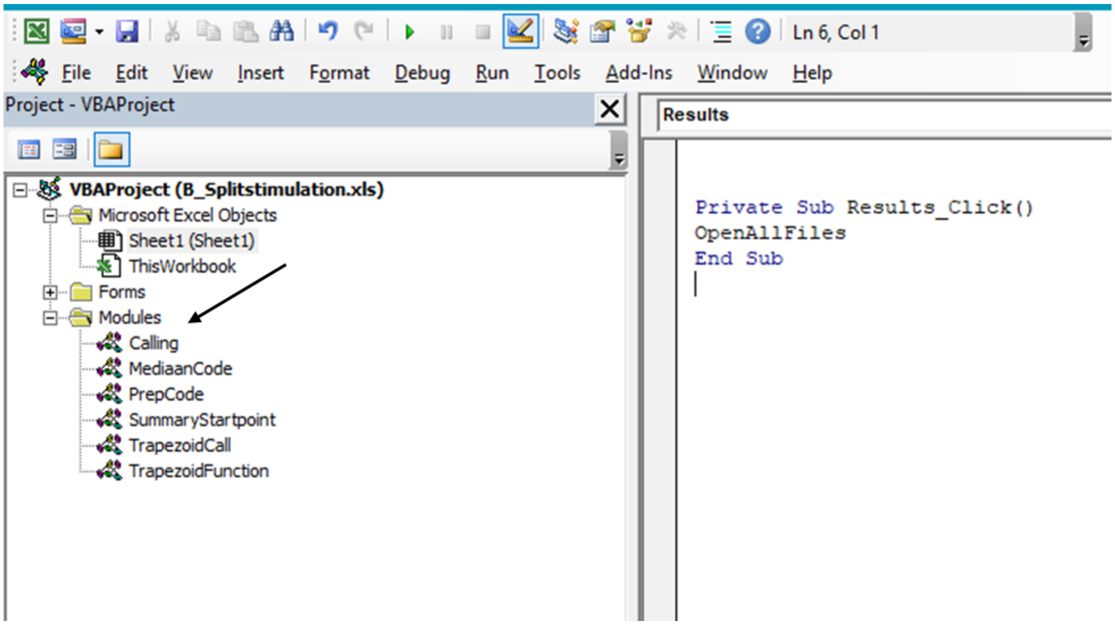

Figure 30

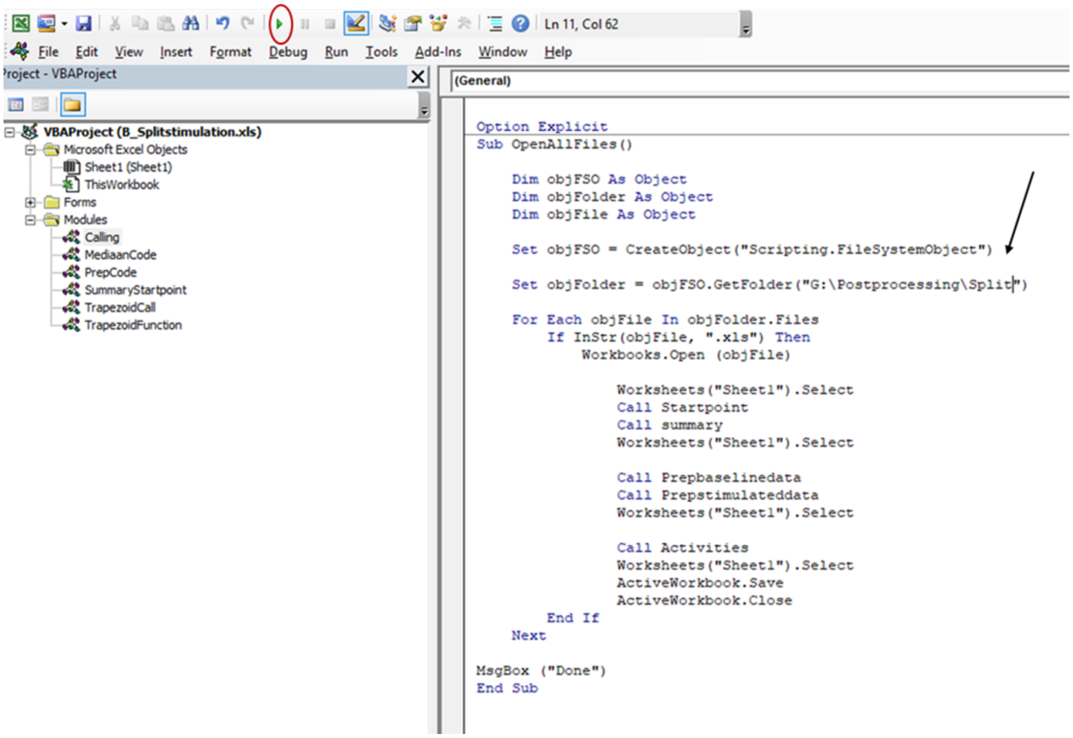

Figure 31

C_Summarizeactivity.m

```
clear all
close all


%define the folder containing the pdata
    % extract pdatafile names
 pparent = 'G:\Postprocessing\Split'; % this is the folder where your split pdata xls files are
[d_file,N_file] = verify_dirlist(pparent,0,'xls');

A=[];
B=nan(1,240);


% % extract stimulated data
% % extractions happens per file
%
for n = 1:N_file

    pfile = fullfile(pparent,d_file(n).name);
    [~,sheet_name]=xlsfinfo(pfile);
    data=xlsread(pfile,sheet_name{1});  %%% change sheet_name{2} if you want to have baseline activities instead

    B=data(end-3,2:241);

    A = [A;B];

end


xlswrite( 'Summarized_activity.xls',A) %% change name as needed for upcoming excel step
```

Command Window

```
>>
```

Figure 32

3.5. Calculate life-and healthspan metrics

From the daily activities per worm obtained in 3.4, you can now generate activity declines for individuals and for populations of worms. Individual activity declines are used by the "***D_Metricscalculation.xlsm***" script to determine LS, HS, TDH and derived H(S)R as defined in Jushaj *et al.*, 2019. For populations, average values can also be calculated. In addition, this script can plot survival and activity decline curves for each studied population.

To use the "***D_Metricscalculation.xlsm***" script, follow these steps:

Step 1. Make sure that you have created a "metrics folder" containing three subfolders: (1) "GTfiles", (2) "Plates" and (3) "Timefiles", as described previously. Copy the activity summary files from section 3.4 to the "Plates" folder. This is the step for which the earlier-mentioned 5-

character limit matters (*e.g.* EXP01, see also "2.2 Differential image analysis"). If desired, the permitted character number for plate naming can (and should) be changed in script modules 2 and 9.

Step 2. In the "Timefiles" folder, a file containing numerical identifiers of days on which data were gathered, should be manually generated (*e.g.* 1,2,3 etc. for days 1,2,3 etc. of adulthood). This is needed to create relevant time identifiers for each activity value. These files should always be named "experimentname-GT-time.xlsx" (*e.g.* EXP01-GT-time.xlsx) and contain an n×1 table (single column) where n is the number of monitoring blocks within the entire observation period. For the data of this tutorial, the column thus sequentially lists relative days on which the data were collected (1, 2, 3, …). It is important that the number of rows of the activity summary files equals n.

> **<u>Note:</u>** it is possible, and advisable, to analyze multiple experiments at the same time, by simply copying multiple activity summary files in the "Plates" folder and creating accompanying 'Timefiles'. The "GTfiles" folder is still empty at this point.

> **<u>Note:</u>** It is advised to save a back-up of the activity summary files in a different location, as "***D_Metricscalculation.xlsm***" changes these by adding headers. Should an error occur while executing this script, *e.g.* a human error where the paths have been incorrectly defined, activity summary files might have already been altered by the addition of headers and now need to be restored to their original format (*i.e.* without headers) when rerunning the script. This may become needlessly labor-intensive and the replacement by the original (backed-up) activity summary files may be far less time consuming.

Step 3. In the '*Developers*' tab, click '*View code*'. The 'calling' module for this script is module 8 (Figure 34). Prior to running the script, at several places in modules 1, 2, 8 and 9, the directory paths of the "GTfiles", "Plates" and "Timefiles" folders need to be specified. Moreover, in modules 5 and 6, the threshold for health can be changed (as is also indicated explicitly in the script).

Step 4. Run module 8 to start the script.

At the end of this script, for each performed experiment, an extra file should have been created in the "GTfiles" folder. In this file, worms will be grouped (per quadrant, as per Figure 1) in Sheet1 and daily activities are replaced by life status (alive or dead) and colored according to health (green: healthy; yellow: unhealthy; no color: not alive). Information in other generated sheets includes the population activity decline, summaries of all LS, HS, TDH, H(S)R values and the survival curves of the populations (Figures 35-39).

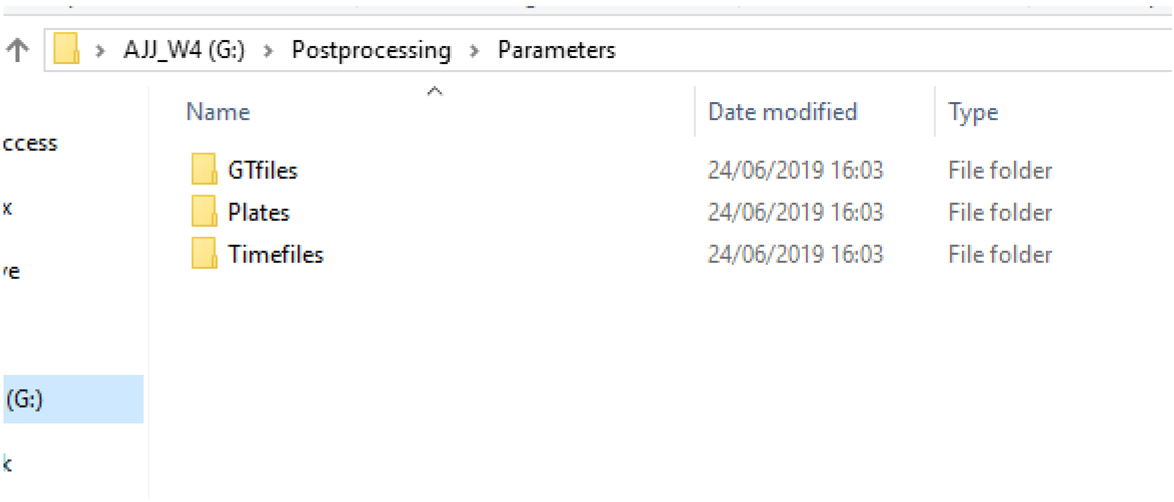

Figure 33

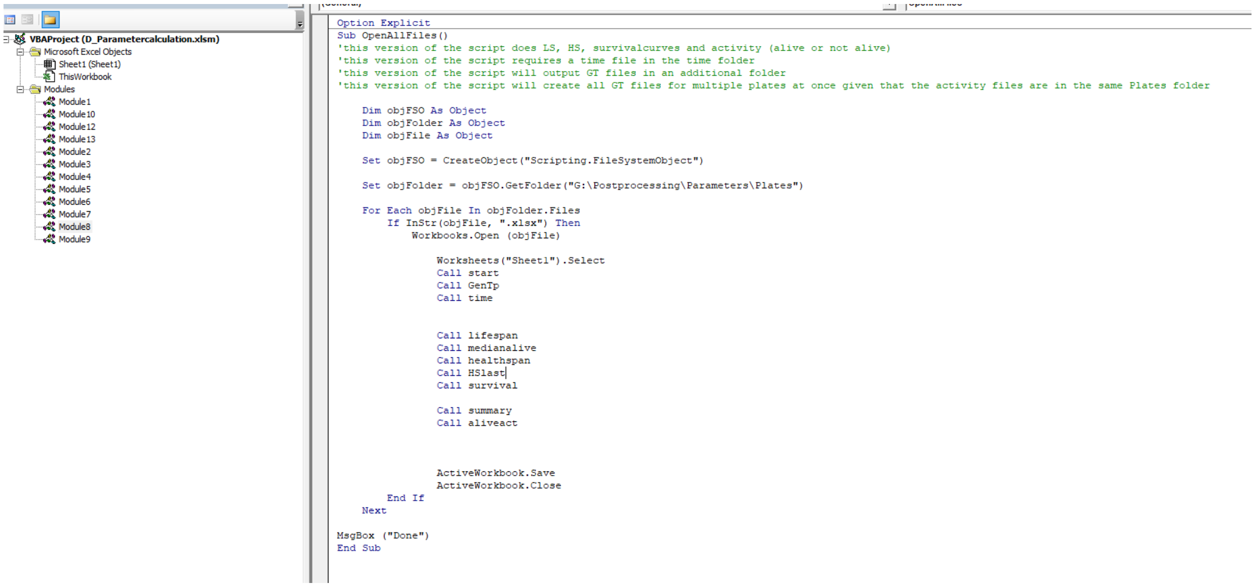

Figure 34

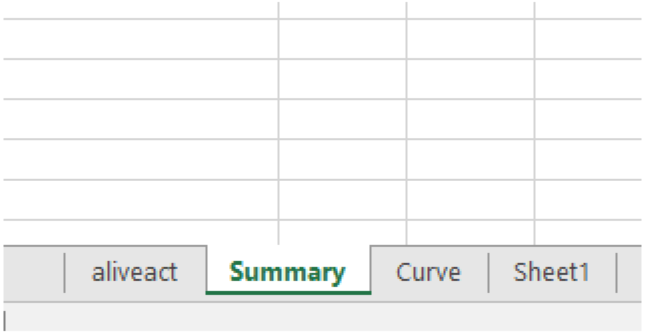

Figure 35

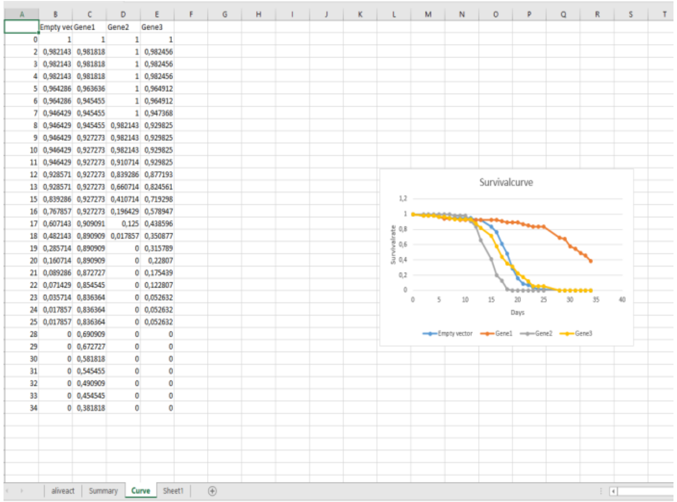

	Empty vec	Gene1	Gene2	Gene3
0	1	1	1	1
2	0,982143	0,981818	1	0,982456
3	0,982143	0,981818	1	0,982456
4	0,982143	0,981818	1	0,982456
5	0,964286	0,963636	1	0,964912
6	0,964286	0,945455	1	0,964912
7	0,946429	0,945455	1	0,947368
8	0,946429	0,945455	0,982143	0,929825
9	0,946429	0,927273	0,982143	0,929825
10	0,946429	0,927273	0,982143	0,929825
11	0,946429	0,927273	0,910714	0,929825
12	0,928571	0,927273	0,839286	0,877193
13	0,928571	0,927273	0,660714	0,824561
15	0,839286	0,927273	0,410714	0,719298
16	0,767857	0,927273	0,196429	0,578947
17	0,607143	0,909091	0,125	0,438596
18	0,482143	0,890909	0,017857	0,350877
19	0,285714	0,890909	0	0,315789
20	0,160714	0,890909	0	0,22807
21	0,089286	0,872727	0	0,175439
22	0,071429	0,854545	0	0,122807
23	0,035714	0,836364	0	0,052632
24	0,017857	0,836364	0	0,052632
25	0,017857	0,836364	0	0,052632
28	0	0,690909	0	0
29	0	0,672727	0	0
30	0	0,581818	0	0
31	0	0,545455	0	0
32	0	0,490909	0	0
33	0	0,454545	0	0
34	0	0,381818	0	0

Figure 36

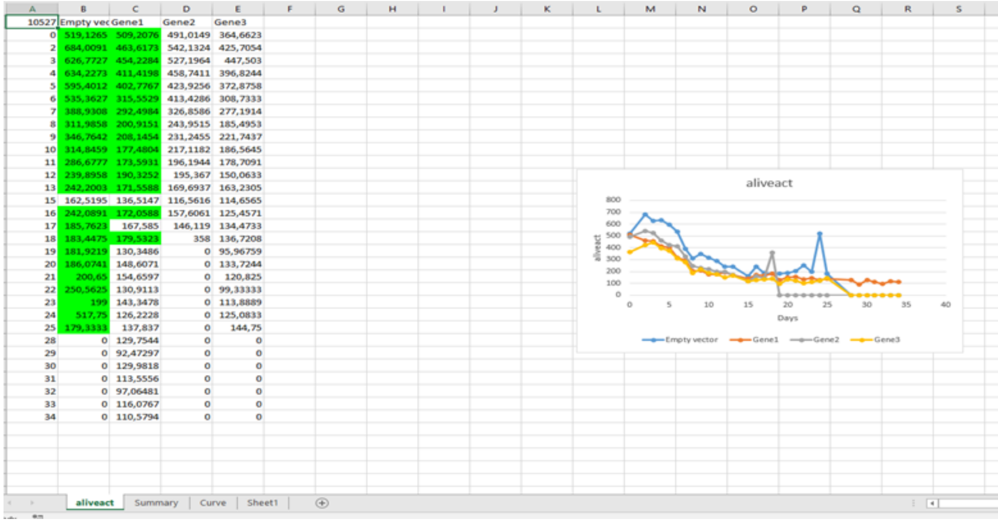

10527	Empty vec	Gene1	Gene2	Gene3
0	519,1265	509,2076	491,0149	364,6623
2	684,0091	463,6173	542,1324	425,7054
3	626,7727	454,2284	527,1964	447,503
4	634,2273	411,4198	458,7411	396,8244
5	595,4012	402,7767	423,9256	372,8758
6	535,3627	315,5529	413,4286	308,7333
7	388,9308	292,4984	326,8586	277,1914
8	311,9858	200,9151	243,9515	185,4953
9	346,7642	208,1454	231,2455	221,7437
10	314,8459	177,4804	217,1182	186,5645
11	286,6777	173,5931	196,1944	178,7091
12	239,8958	190,3252	195,367	150,0633
13	242,2003	171,5588	169,6937	163,2305
15	162,5195	136,5147	116,5616	114,6565
16	242,0891	172,0588	157,6061	125,4571
17	185,7623	167,585	146,119	134,4733
18	183,4475	179,5323	358	136,7208
19	181,9219	130,3486	0	95,96759
20	186,0741	148,6071	0	133,7244
21	200,65	154,6597	0	120,825
22	250,5625	130,9113	0	99,33333
23	199	143,3478	0	113,8889
24	517,75	126,2228	0	125,0833
25	179,3333	137,837	0	144,75
28	0	129,7544	0	0
29	0	92,47297	0	0
30	0	129,9818	0	0
31	0	113,5556	0	0
32	0	97,06481	0	0
33	0	116,0767	0	0
34	0	110,5794	0	0

Figure 37

A	B	C	D	E	F	G	H	I	J	K	L	M	N	O	P	Q	R	S	T
10527		Worm 1	Worm 2	Worm 3	Worm 4	Worm 5	Worm 6	Worm 7	Worm 8	Worm 9	Worm 10	Worm 21	Worm 22	Worm 23	Worm 24	Worm 25	Worm 26	Worm 27	
	0	Alive	Alive	Alive	Alive	Alive	Alive	Alive	Alive	Alive	Alive	Alive	Alive	Alive	Alive	Alive	Alive	Alive	
	2	Dead	Alive	Alive	Alive	Alive	Alive	Alive	Alive	Alive	Alive	Alive	Alive	Alive	Alive	Alive	Alive	Alive	
	3	Dead	Alive	Alive	Alive	Alive	Alive	Alive	Alive	Alive	Alive	Alive	Alive	Alive	Alive	Alive	Alive	Alive	
	4	Dead	Alive	Alive	Alive	Alive	Alive	Alive	Alive	Alive	Alive	Alive	Alive	Alive	Alive	Alive	Alive	Alive	Dead
	5	Dead	Alive	Alive	Alive	Alive	Alive	Alive	Alive	Alive	Alive	Alive	Alive	Alive	Alive	Alive	Alive	Alive	Dead
	6	Dead	Alive	Alive	Alive	Alive	Alive	Alive	Alive	Alive	Alive	Alive	Alive	Alive	Alive	Alive	Alive	Alive	Dead
	7	Dead	Alive	Alive	Alive	Alive	Alive	Alive	Alive	Alive	Alive	Dead	Alive	Alive	Alive	Alive	Alive	Alive	Dead
	8	Dead	Alive	Alive	Alive	Alive	Alive	Alive	Alive	Alive	Alive	Dead	Alive	Alive	Alive	Alive	Alive	Alive	Dead
	9	Dead	Alive	Alive	Alive	Alive	Alive	Alive	Alive	Alive	Alive	Dead	Alive	Alive	Alive	Alive	Alive	Alive	Dead
	10	Dead	Alive	Alive	Alive	Alive	Alive	Alive	Alive	Alive	Alive	Dead	Alive	Alive	Alive	Alive	Alive	Alive	Dead
	11	Dead	Alive	Alive	Alive	Alive	Alive	Alive	Alive	Alive	Alive	Dead	Alive	Alive	Alive	Alive	Alive	Alive	Dead
	12	Dead	Alive	Alive	Alive	Alive	Alive	Alive	Alive	Alive	Alive	Dead	Alive	Dead	Alive	Alive	Alive	Alive	Dead
	13	Dead	Alive	Alive	Alive	Alive	Alive	Alive	Alive	Alive	Alive	Dead	Alive	Dead	Alive	Alive	Alive	Alive	Dead
	15	Dead	Alive	Alive	Alive	Alive	Dead	Alive	Alive	Alive	Alive	Dead	Alive	Dead	Dead	Alive	Alive	Alive	Dead
	16	Dead	Alive	Alive	Alive	Alive	Dead	Alive	Alive	Alive	Alive	Dead	Dead	Dead	Dead	Alive	Alive	Alive	Dead
	17	Dead	Alive	Alive	Alive	Alive	Dead	Alive	Alive	Alive	Alive	Dead	Dead	Dead	Dead	Alive	Dead	Alive	Dead
	18	Dead	Alive	Alive	Alive	Alive	Dead	Alive	Alive	Alive	Dead	Dead	Dead	Dead	Dead	Alive	Dead	Alive	Dead
	19	Dead	Alive	Dead	Dead	Alive	Dead	Dead	Alive	Alive	Dead	Dead	Dead	Dead	Dead	Alive	Dead	Dead	Dead
	20	Dead	Dead	Dead	Dead	Dead	Dead	Dead	Alive	Alive	Dead	Dead	Dead	Dead	Dead	Alive	Dead	Dead	Dead
	21	Dead	Dead	Dead	Dead	Dead	Dead	Dead	Alive	Alive	Dead	Dead	Dead	Dead	Dead	Dead	Dead	Dead	Dead
	22	Dead	Dead	Dead	Dead	Dead	Dead	Dead	Alive	Alive	Dead	Dead	Dead	Dead	Dead	Dead	Dead	Dead	Dead
	23	Dead	Dead	Dead	Dead	Dead	Dead	Dead	Alive	Dead	Dead	Dead	Dead	Dead	Dead	Dead	Dead	Dead	Dead
	24	Dead	Dead	Dead	Dead	Dead	Dead	Dead	Alive	Dead	Dead	Dead	Dead	Dead	Dead	Dead	Dead	Dead	Dead
	25	Dead	Dead	Dead	Dead	Dead	Dead	Dead	Alive	Dead	Dead	Dead	Dead	Dead	Dead	Dead	Dead	Dead	Dead
	28	Dead	Dead	Dead	Dead	Dead	Dead	Dead	Dead	Dead	Dead	Dead	Dead	Dead	Dead	Dead	Dead	Dead	Dead
	29	Dead	Dead	Dead	Dead	Dead	Dead	Dead	Dead	Dead	Dead	Dead	Dead	Dead	Dead	Dead	Dead	Dead	Dead
	30	Dead	Dead	Dead	Dead	Dead	Dead	Dead	Dead	Dead	Dead	Dead	Dead	Dead	Dead	Dead	Dead	Dead	Dead
	31	Dead	Dead	Dead	Dead	Dead	Dead	Dead	Dead	Dead	Dead	Dead	Dead	Dead	Dead	Dead	Dead	Dead	Dead
	32	Dead	Dead	Dead	Dead	Dead	Dead	Dead	Dead	Dead	Dead	Dead	Dead	Dead	Dead	Dead	Dead	Dead	Dead
	33	Dead	Dead	Dead	Dead	Dead	Dead	Dead	Dead	Dead	Dead	Dead	Dead	Dead	Dead	Dead	Dead	Dead	Dead
	34	Dead	Dead	Dead	Dead	Dead	Dead	Dead	Dead	Dead	Dead	Dead	Dead	Dead	Dead	Dead	Dead	Dead	Dead
	34	0	19	18	18	19	13	18	25	22	17	6	15	11	13	20	16	18	
		0	12	7	12	16	12	14	24	14	10	4	10	9	8	15	11	14	0
			18	9	18	18	13	18	25	22	12	4	15	11	13	17	16	17	

aliveact | Summary | Curve | Sheet1

Figure 38

A	B	C	D	E	F	G	H	I	J	K	L
10527											
Empty vector-LS	0	19	18	18	19	13	18	25	22	17	6
Empty vector-HStotaldays	0	12	7	12	16	12	14	24	14	10	4
Empty vector-HRtotaldays		0,631579	0,388889	0,666667	0,842105	0,923077	0,777778	0,96	0,636364	0,588235	0,666667
Empty vector-HSlastday		18	9	18	18	13	18	25	22	12	4
Empty vector-HRlastday		0,947368	0,5	1	0,947368	1	1	1	1	0,705882	0,666667
Gene1-LS	34	34	34	22	25	34	34	34	34		34
Gene1-HStotaldays	17	29	26	16	19	28	16	21	12		24
Gene1-HRtotaldays	0,5	0,852941	0,764706	0,727273	0,76	0,823529	0,470588	0,617647	0,352941		0,705882
Gene1-HSlastday	34	34	30	21	25	34	28	33	25		34
Gene1-HRlastday	1	1	0,882353	0,954545	1	1	0,823529	0,970588	0,735294		1
Gene2-LS	15	18	15	13	13	12	12	17	12	13	11
Gene2-HStotaldays	12	16	14	11	12	12	12	11	8	13	10
Gene2-HRtotaldays	0,8	0,888889	0,933333	0,846154	0,923077	1	1	0,647059	0,666667	1	0,909091
Gene2-HSlastday	13	18	15	13	12	12	12	15	10	13	11
Gene2-HRlastday	0,866667	1	1	1	0,923077	1	1	0,882353	0,833333	1	1
Gene3-LS	16	25	19	22		20	13	12	15	4	15
Gene3-HStotaldays	11	16	13	17		12	9	9	6	4	14
Gene3-HRtotaldays	0,6875	0,64	0,684211	0,772727		0,6	0,692308	0,75	0,4	1	0,933333
Gene3-HSlastday	13	25	17	21		12	10	10	6	4	15
Gene3-HRlastday	0,8125	1	0,894737	0,954545		0,6	0,769231	0,833333	0,4	1	1

aliveact | Summary | Curve | Sheet1

Figure 39

3.6. Working with life- and healthspan quantifiers

Some wells may not pass automated quality criteria (*e.g.* empty wells), leading to missing values. Because these may skew the analysis (*e.g.* they should not contribute to calculations of averages etc.), these are automatically removed. For these worms, lifespan is not calculated and results in a blank "cell" in the excel file. However, because the way TDH and HS are calculated by the script, *i.e.* by counting 'healthy' days, these missing worms will be assigned a TDH and HS of "0". This "0" eventually contributes to the calculation of average TDH/HS and H(S)R, and therefor needs to be removed, resulting in a blank "cell" as is the case for lifespan. Blank "cells" are ignored in excel when averages are calculated and will therefore not affect the results.

To do this, use the "***E_getridofzeros.xlsm***" script, where you will need to change the folder path in the 'calling' module (module 2), as indicated in the script, and run this module.

The main goal of this analysis is to probe for possible significant effects of interventions (here: Genes 1-3) on the LS, HR (and IA – see section 3.7) of the studied worm populations. In order to interpret results, data needs to be grouped per studied genotype.

The following step is required:

Step 1. Open "***F_LSHScopy.xlsm***" and change the location of the "LSHSdata" folder as noted in modules 1, 2 and 3 of the script. Run the 'calling' module 2.

3.7. Calculate integrated activity and compare worm populations

A final metric for evaluation of potential differences between genotypes is integrated activity, calculated as the definite integral of the area under the population activity curve (see also Jushaj *et al.*, 2019 for more information).

To calculate this value:

Step 1. Open "***G_Activcopy.xlsm***" and change the locations as indicated by the script. Run the 'calling' module 3. This will result in a new excel file, 'EXP01-activ.xlsx'(Located in the "Activities" folder), that contains the activity decline of the different populations in your WorMotel plate.

If the name of your experiment is solely a number, running the "***H_ReplaceRA1.xlsm***" script is required. For this, simply run module 2 of this script. This is not needed for all other names (including mixed ones, such as EXP01).

Step 2. Run "***I_Comparativeanalysis.m***" in Matlab® and indicate in the upcoming interfaces the location/path of the "Activities", "LSHSdata" and "normalised coordinates" folder. The script calculates the average LS and HR values per studied population. Additionally, it calculates the integrated activity, as described by Jushaj *et al.*,2019. These values are then normalized to that of the internal control, resulting in each population being characterized by its normalized average LS, HR and IA.

The script outputs *.xlsx* files (located in the 'normalized coordinates' folder and called 'EXP01_nLSHRAUC.xlsx') containing the normalized average LS, HR and IA for the four studied conditions in the order:

nLS_{IC} nHR_{IC} nIA_{IC} nLS_{G1} nHR_{G1} nIA_{G1} nLS_{G2} nHR_{G2} nIA_{G2} nLS_{G3} nHR_{G3} nIA_{G3}

A summarizing file (located in the 'normalized coordinates' folder and called 'alldata_nLSHRAUC.xlsx') is also generated and contains the same information as found in 'EXP01_nLSHRAUC.xlsx'. This file can be practical when analyzing multiple WorMotel plates

simultaneously, as it will list the results of the individual '_nLSHSAUC.xlsx' files (descending rows in alphabetical order of the assigned experiment name).

In the "LSHSdata" folder, two additional files are created: (1) 'EXP01_meannormLSHR.xlsx' and (2) 'EXP01_nAUC.xlsx' containing respectively the LS&HR data and the IA data for the analyzed experiment/plate.

4. Conclusions

This tutorial allows you to quantify and evaluate the effects of interventions on the lifespan and health of *C. elegans* using the WorMotel system. You should now be able to interpret the effect of the intervention on the average lifespan, health ratio and integrated activity of studied populations. In addition, you are equipped with survival and activity decline curves. For statistical analysis of your data, LS and HR of individual worms are grouped in assigned populations and can easily be extracted (*e.g.* 'LSHS' file in the "LSHSdata" folder. It is to be noted that for a single plate, no statistical analysis can be performed on IA, as one WorMotel experiment results in only one IA value per studied population.
