# Supplementary material for: Optimized criteria for locomotion-based healthspan evaluation in C. elegans using the WorMotel system
Source: PLoS One. 2020 Mar 3;15(3):e0229583. doi: 10.1371/journal.pone.0229583 (PMC7053758; doi:10.1371/journal.pone.0229583)
Supplement: Attachment — Submitted filename: Response to Reviewers.pdf [file pone.0229583.s021.pdf]

Reviewer 1

General remarks

Summary: The paper focuses on optimizing the use of movement-based data from the WorMotel system to better evaluate healthspan. They do this by first evaluating four different statistics of single worm movement before choosing the one they claim is the least variable over independent experiments (peak activity). They then measure "total days of health" and healthspans of daf-2 and daf-16 RNAi animals, both of which are based on varying thresholds of peak activity compared to wildtype. They further analyze these data by using various ratios and integrals to develop further differences between the phenotypes of the animals and to most robustly capture differences in their healthspans.

The principal of these experiments, developing and evaluating statistics to measure worm movement in high-throughput automated systems, is worthwhile. Much of the approach is warranted and well-described. However, some of the tools and many of the quantitative "cutoffs" and measurements are not properly justified. Also, the text does not take confounding issues into account, relying solely upon quantitative movement measurements with little regard to what they might mean and what they might really be measuring. As a whole, the manuscript takes what could be interesting data and makes many assertions that are not well-justified toward the main subject (healthspan/healthy aging) and are not put into a context that makes sense beyond statistical analysis.

> *We thank the reviewer for conceptually appreciating our undertaking and hope the revised version of our manuscript is now within expectations. We have addressed all comments in more detail below:*

Major concerns

While pixels changed is a nice quantifiable readout, it is unclear what this represents. It would not be difficult to use the recordings to produce a qualitative corollary (fast/slow movement across plate, partial paralysis, movement in place etc.) and to then quantify these movements as more reliable readouts of movement with pixels changed. The use of an arbitrary quantitative cutoff when qualitative data can be added to it seems like a lost opportunity.

> ***Addressed in lines 329-363****: qualitative vs quantitative measurements are now included.*

Calling daily peak activity more robust ignores what these measurements are supposed to take into account. Health is definitely not judged by peak activity (e.g. sprinting), and just because a measurement creates quantitative spread, it doesn't make it the best measurement. None of these things conclusively measure health.

> *It is not our intention to claim that our locomotion-based readout would be the sole, definitive measurement for organismal health. Several readouts of ageing have been described for C. elegans, such as oxidative and heat stress resistance, pharyngeal pumping and autofluorescence* [1], *vulval integrity* [2], *intestinal atrophy* [3], *muscle integrity and yolk production* [4], *amongst which locomotion is the most attractive proxy for automated analysis by medium-to-high throughput screening platforms, e.g.* [5]. *Here, our goal is to determine reliable criteria for selection of candidate interventions in WorMotel-based screening efforts of considerable throughput, where fast selection of candidates of interest is imperative. Obviously, for a detailed understanding of their impact and mode of action, selected candidates would then require a more in-depth study of health during aging.*

Please note that all parameters discussed in the manuscript measure the animal's response to blue light. These are therefore not readouts of the animal's inherent maximal ability to sprint, 'peak activity' is rather to be interpreted as an animal's initial response to the stimulus.

Why is it necessary to convert activity to a single value per day? Why not more frequently? Less frequently? Why not multiple values? It seems like they are just trying to make it simple for simplicity and not for a well-justified reason.

Obviously, adjustments to the measurement scheme would be possible. It is customary to perform daily measurements in C. elegans ageing research, and we did not evaluate the effect of reducing the imaging frequency even further. Previously, Petrascheck et al. [6] *reported that less frequent measurements (every 2-5 days) are possible, but require increased numbers of animals to achieve sufficient statistical power. With 60 worms per condition per plate, daily imaging of WorMotel plates can detect a change of 6% with a power of 80% for confidence intervals of 0.95 (Fig R1). Our earlier work also reports on higher imaging frequencies (twice per day,* [7]*), which did not affect experimental conclusions. (1) Because of this, and because increasing imaging frequency (2) comes at a direct cost of throughput, and (3) increases the cumulative dose of blue light received by the animals, daily imaging is the most sensible choice.*

The conversion of post-stimulation (10 min) behavior to a single value per day, addresses the discontinuous nature and time scales of the collected data. Such an operation is standard practice for any repeated testing of animals (e.g. learning indices over time, etc.). One of the aims of the manuscript is to address a lack of standard for such operations when evaluating stimulated activity over ageing, and to explain analytical choices.

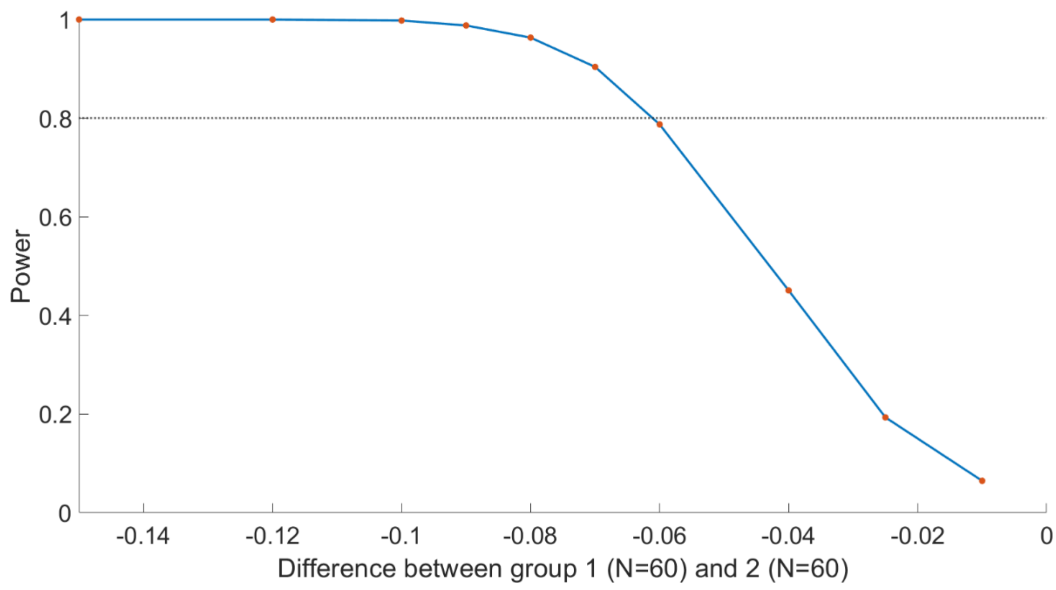

Figure R1. Monitoring 60 animals per group achieves sufficient power to detect changes of 6%. *WorMotel imaging can detect effects larger than 6 %, at confidence intervals of 0.95, for a standard power cutoff of at least 80%.*

All of the choices presented for single value readouts seem to have more random variation than one would like, reflecting higher day-to-day oscillations and thus challenges in the readout. This is a function of the complexity of complex organisms.

*We agree with the reviewer that the observed day-to-day variation is high, and can be a result of multiple factors - including sparse sampling, small technical variation, but also worm biology and the multifactorial nature of 'complexity of complex organisms'. A more balanced discussion of variation and different assumptions that go with it, is now part of the revised manuscript (**lines 257-279**).*

Is peak response really the maximum ability to move? Or does it also have to do with sensitivity to the blue light changing with age and a number of other unknown factors? None of the other potential factors are mentioned or accounted for.

Addressed in lines 466-472. *We apologize for not having clarified this sufficiently in the initial draft of the manuscript. The measured activity is an integrative readout of neuromuscular health. With respect to sensing/moving alterations: abilities of animals to differentially maintain either, are interesting in light of healthy ageing. This is now more clearly explained in the revised manuscript (**lines 460-465**).*

"Smoother curves lead to more robust assessment." They may lead to more separation, but that doesn't make it necessarily correct. It is looking at different things, some of which have different variation, and none of which are looked at for why they vary and thus why they might be better or worse indicators of health. It is impossible to say what is better or worse at measuring health, only that some are statistically more separable.

Addressed in lines 257-271. *We agree and have adjusted the text to better reflect the uncertainty on sources of variation, and possible interpretations of it.*

What is a "non-optimal activity?" How can one assess this? What does this mean? Without qualitative measurements this is a completely arbitrary cutoff with no real meaning.

Addressed in lines 328-355. *Qualitative measurements are now also included, and blinded experiments were the basis for determining a cutoff.*

What exactly does "total days of health" tell you? They define the parameter, but how does it contribute to a better description of the health of the animal? It seems they use it primarily because it is less than the healthspan (in numerical quantity) resulting in better resolution of the health ratios they utilize later in the paper. If the healthy days don't have to be consecutive, what does it mean when a worm doesn't have a "healthy" day, but resumes being healthy the following day? HR "outperforms" HSR as a distinguishing health metric? What does this mean? Arbitrarily

creating increased differences is not outperforming. It is not justified why TDH is a useful metric, what it really means, or whether it has any place in measurements.

> ***Addressed in lines 387-401.*** *HS is more likely to falsely overestimate animal health, than TDH. The revised manuscript contains additional information explaining the performance of TDH vs HS values for different prototypical ageing profiles (added as* ***Figure S12****). In deciding on health, TDH is the more conservative (fewer false positives) metric.*

"The WorMotel setup uses blue light to stimulate the animals therefore peak activity reflects the maximal ability of an animal to move, and it is evaluated over its lifetime" This may be true in young worms, but is not known in older individuals who lose not only the ability to move, but to perceive things such as blue light.

> ***Addressed in lines 469-471.*** *The reviewer is completely correct (and even in younger animals, this is not necessarily the case). This was an unfortunate phrasing, which is adjusted in the revised manuscript. We* [7] *previously showed that lifespans of blue-light stimulated animals are comparable to those of animals stimulated by touch (the used golden standard in manual assays - arguably also a sensory stimulus, albeit a different one). This showed that blue light stimulation can be used to study lifespan in C. elegans. With regards to healthy ageing, either possible source listed by the reviewer would lead to a selection of the intervention (scored as less healthy overall), which would be in line with expectations: a fast, first selection of interventions expected to affect healthy ageing.*

Minor concerns:

Figure 3: Peak activity and 99th percentile appear do have the lowest variation but no statistics that they are significantly different than median or integrated activity are given.

*Please refer to **Table S2** (Kruskal-Wallis).*

Figure 6B, D: B has no error bars (or maybe they're just really small) while D has huge error bars. In general, dividing both TDH and healthspan by the same value (lifespan) is plotting the same information on a different scale. This seems unnecessarily redundant since the trends are going to be the same.

Addressed in figures 6 and 8. *We thank the reviewer for having noticed a mistake in the error bars. In addition, figures have been adjusted according to this reviewer's request.*

Line 315: They propose 100 seconds as the "ideal compromise" for the time intervals, but if they identified 60 – 300 seconds a finer gradient would be necessary to find a more optimal time instead of just taking the middle time point between the two.

Addressed *by the additional evaluation of time-intervals 80, 150, 200 and 250 seconds in the revised manuscript.*

Figure 7: They never actually define the acronym IA in the paper. A) The variation in their individual daf-2 RNAi experiments makes it hard to determine the significance of the points compared to WT. B) It would seem that the daf-16 RNAi animals are healthier but move slightly less than WT over their lifespans, while the daf-2 RNAi animals are less healthy but move more than WT over their lifespans.

The daf-2 RNAi animals are healthier (i.e. they move more) at later stages in life (the "twilight" period) than WT, but since their activity is below the threshold chosen by the authors, daf-2 RNAi animals are considered to be less healthy than WT. This illustrates the problem with arbitrary thresholds: depending where they are set you can get either answer.

> ***Addressed and discussed in lines 387-401*** *of the revised manuscript, bearing in mind the additional qualitative analysis upon which the choice of threshold now builds.*

Figure S6: It appears the plots are reversed compared to what they describe in the caption.

> *This was a correct observation, however, this figure is no longer included in the revised manuscript.*

Discussion:

Line 355: "…a wide variety of interventions can be tested…" Only one type of was tested here. This seems like an overreach.

> ***Addressed in lines 423-424.*** *We agree and have adapted the text.*

Reviewer 2

General remarks

SUMMARY

The authors use data from the previously described "WorMotel" system to evaluate metrics for assaying health and healthspan. In particular, the authors compare N2, daf-2(RNAi) and daf-16(RNAi) for health effects under a variety of metrics to identify an "optimal" scheme for measuring individual and population health (according to several metrics the authors have chosen). The authors find that peak activity across measurement intervals of 60-300 sec. provide the least variable measurements of movement among those considered. They further argue that two metrics provide useful and complementary information about health: indivdual health ratios (a ratio of a healthspan-like metric to lifespan) and population-level integrated activity.

IMPRESSION

There is great experimental and conceptual inconsistency within the field regarding how to evaluate "healthspan" in C. elegans. The current study provides a useful starting point for a much-needed discussion about experimental design and data analysis.

Before that, however, there are several points within the study where the authors need to clarify methodological details, and explicate some of the logic by which they draw their conclusions. In particular, the criteria by which the authors evaluate "optimal" metrics are rather ad hoc and deployed with little justification or statistical backing. More explanation here will be crirical.

In related matters, the authors reasoning in justifying their choice of health measures is somewhat circular in places. The main logic appears to be that "a good healthspan metric should distinguish

daf-2, daf-16, and control conditions" -- which presumes that healthspan is actually different in those cases. What if the "true" healthspan (or normalized healthspan ratio) is actually the same in daf-16 vs. N2? There's no a priori reason to assume that the "right" metric is one that separates those conditions. At a minimum these caveats need to be carefully discussed.

The writing is also somewhat loose and informal throughout, and in places overly vague regarding specific experimental details. Nevertheless, with a little tightening of both the writing and the logic of the data analysis -- and perhaps dialing some of the conclusions back to what is supported by the data -- this will be a useful contribution to the literature.

We thank the reviewer for sharing an interest in opening the discussion on measuring health and healthspan in C. elegans. Our revised manuscript addresses the concerns listed above, as detailed in the point-by-point responses below:

MAJOR CONCERNS

In the abstract, the authors refer to RNAi against daf-2 and daf-16 (vs. empty-vector control) as a "wide range of conditions". Indeed, throughout the work the authors simply assume that any findings based on analysis of these three conditions will inherently generalize to other (non-IIS-perturbing) conditions. This is a bit of a stretch, and the authors need to be much more careful with claims of generality after examining only IIS-pathway perturbations.

Addressed in lines 225-226 *et al. We agree with the reviewer and have adapted the text accordingly.*

Many of the methods are under-described, especially for a manuscript that aims to propose a canonical analysis scheme.

We hope the revised version of the manuscript is now in line with the reviewer's expectations.

The authors need to describe precisely how image pixels are turned into numerical scores. I assume the image immediately before stimulation is compared to images at different intervals after stimulation. (Or are the differences calculated not for t=0 vs. t=n, for all n, but for t=n vs. t=n+1? This matters, obviously.) Next, I assume a pixel-wise difference image is calculated for whichever pair of images is under consideration. But how is that image then converted into a single difference score? The median absolute pixel difference? The root mean squared pixel difference? The sum of absolute differences? The count of pixels that are different by a certain threshold? (If the latter, how is the threshold chosen? And how does the threshold choice influence subsequent conclusions regarding healthspan?)

If a sum-of-differences type of metric is used (rather than e.g. a count-of-above-threshold-differences), the authors discuss the caveat that conditions that change that pixel intensity distribution of worms (i.e. produce individuals that are either more clear or darker or more mottled than WT) will naturally generate different pixel differences for the same amount of total movement. This is also a problem for thresholded counts, but may be less severe depending on the threshold employed. Overall, these details matter, and they need to be explicitly described in the methods (rather than be left implicit in a matlab script) and the choices / trade-offs should ideally be justified if possible.

Addressed in lines 112-118. *While the reviewer's questions are already addressed in* [7], *we also expanded the methodology of our manuscript to briefly include this information. The 5 pixel difference threshold (i.e. below 5 pixels of difference = dead) and the binary*

threshold of 0.25 to minimize image noise (see Methods) are based on empirical evaluation by [7] *to minimize false positives and false negatives.*

Given a pixel-difference score as a function of time post-stimulation, the authors next propose several ways to summarize that as a single number for each individual. Of these, the "peak activity" score needs to be described and justified better. What does "average of the 95th to 99th percentiles" mean? Is it the mean of all values within the range defined by the 95th to 99th percentiles? Or just (95th-percentile-value + 99th-percentile-value)/2? More generally, what is the point of using both a percentile and an arithmetic mean? The choice is justified as saying it "should be slightly less prone to outliers or noise", but I'm not sure on what statistical basis one might conclude that a mean (which is outlier prone) of a percentile (which is more robust to outliers) would be better than just e.g. using the 97.5th percentile or whatever. Especially given that this mathematically-weird metric is the one the authors recommend later, a little more description / justification is warranted.

Addressed in lines 131-132. *Because these percentiles are not calculated based on populations of the same aimed-for measurement (i.e. no repeated measurements), but based on the distribution of temporally spaced data collected of a single animal, the assumption that high percentile values are resistant to outliers, is false. Also, because these data are not normally distributed, the mean of all values in the 95-99th percentiles does not equal the 97,5th percentile.*

For each 10 mins post-stimulation activity 'trace' (bear in mind this is a pixel difference plot, no high-resolution tracking of locomotion is performed) of a single animal, the 99th percentile reflects the top of the curve (which varies substantially between animals/conditions and over age, and can be outlier-prone). The peak activity is a

compromise between aiming to measure the maximal response while buffering the effect of possible outliers by averaging the top 5% of measured values. In ideal circumstances, peak and maximal activity should correlate perfectly. As can be seen in ***Figure S2A****, this is generally true, showing that most of the time, either value captures the full dynamic range of the animal's response to blue light.*

The overall variation score is not clearly defined. Presumably the brackets in the denominator of the numerator of the overall formula represent the expectation (mean) of the day-to-day absolute changes. But what is the mean over -- is it a mean of all individuals at that day? A mean of all days for that individual?

Next, in the results, this is described as a sum of changes "in percent", but it's not clear that the formula really is calculating percentage changes in any meaningful way. A percent (or fractional) change would generally be calculated something like |a_i - a_i+1| / |a_i|, or similar. (I.e. change / baseline, rather than the current formula, which is individual-change / population-mean-change.)

More generally, this score is rather ad hoc, and the specific choice isn't particularly well justified. In particular, given that some degree of activity-score changes over time is expected (as aging happens, scores decline), clearly an optimal health score isn't just one that minimizes all changes over time!

The authors are correct in their desire for a metric that changes smoothly, rather than one that is noise-prone and gives jagged curves over time. But the authors' proposed variation score doesn't just penalize jagged curves -- it penalizes any curve that isn't completely flat. That is, the only way to minimize the proposed total variation score is for a health measure to give a completely flat, zero-slope line. So, broadly, the key metric for this whole manuscript isn't really measuring what

the authors want it to measure (or claim it is measuring). Intuition: jaggedness is a second-derivative (and higher derivatives) property, but the score is just examining first derivatives.

A better approach might be to minimize day-to-day changes with reference to the overall population trend. Something more like $|a_i/\mathrm{mean}(a_i) - a_{i+1}/\mathrm{mean}(a_{i+1})|$ (where the mean is across all individuals at that timepoint), for example. A good health measure could produce zero on this variation score and still allow for smooth changes in health over time.

Even that's pretty ad hoc, however, and would confounded by changes in the population variance in activity scores over time. So the real right answer is for the authors to look to the existing statistical toolkit, rather than reinventing their own measures of variation. In particular, if the authors wish to measure variation, then statistical variance would be an obvious choice. But one wouldn't want to measure the variance of the a_i since, as above, those scores are expected to change over time. A more principled approach would be to calculate each individual's movement z-score as a function of time i.e. $z_i = (a_i - \mathrm{mean}(a_i)) / \mathrm{std}(a_i)$ (where the mean and std are across all individuals at that timepoint), and then calculate the variance of the z_i scores across all timepoints for that particular individual. The "smoothest" activity measure would be the one with the least variance in z-scores over time.

Note also the "peak activity" metric is truly the best and most robust, one might hope that it would perform best across a panel of different metrics, such as all those suggested above. But if each different variation score yields a different choice of "best metric", one might become skeptical of the whole project.

Remarks have been addressed in Materials and Methods, as well as in the Result section (lines 266-279).

The reviewer is right in observing that flat curves will have lower 'overall variation', however, all curves of the same animal (i.e. based on median/integral or peak/maximal) report on the same gradual decline. Because we compare these different metrics for the same individuals, minimizing overall variation does minimize jaggedness.

A "5 pixels changed" threshold is used to define lifespan (line 146-7 and 265-6), but there is no discussion of (i) how this threshold was chosen, (ii) over what time interval it is computed (since the authors note that longer time intervals increase sensitivity to movement), (iii) how sensitive lifespan estimates are to that specific threshold.

This threshold was determined by [7], *as a suitable cutoff for noise (e.g. light flickering). In this work, lifespans calculated based on this threshold are shown to match manual assessment of lifespan, supporting the validity of this choice. This is now explicitly referenced in* ***line 167*** *of the revised manuscript.*

Regarding the different activity metrics (peak, median, etc.), how much do these distinctions actually matter? Do any of the conclusions from the later sections actually depend on using peak/99th percentile, or are the qualitative results the same if median or integral was employed? For that matter, how well correlated are all of these measures with one another? Perhaps this is all a bit of a distinction without a difference, if all the measures are highly correlated...

Addressed in lines 251-256.

Regarding the determination of an appropriate healthspan descriptor (HR vs. HSR) + threshold (lines 273-299), the logic/language in this section is pretty unclear throughout.

Consider making these paragraphs into a new section altogether (e.g. "determining ideal metrics for health").

It would be useful to clearly state ahead of time that the decision of interest involves choosing HR vs. HSR (as opposed to TDH vs. HS, and separate from just determining the threshold).

lines 273-289 and lines 290-299: the authors appear to be making the assumption that the ideal descriptor and threshold should (i) distinguish among daf-2, daf-16, and WT, and (ii) be consistent across experiments. The authors should state these criteria explicitly and justify them. In particular, as above, this introduces some degree of circular reasoning. If the authors chose their health metric based on how well it can distinguish daf-2 and daf-16 from WT, then some reader of this paper would be on very shaky ground to then use that metric to try to learn anything about the health effects of IIS by using such a metric.

lines 292-295: "at very high thresholds, nTDH reflects the intrinsic activity of the young worm populations (vs control levels), whereas at low thresholds, this value approaches longevity (Fig S4 and S5). The optimal threshold region can be found in between these two, where HR (=TDH/LS) reflects health". The authors' argument seems to be that because the extremes of threshold ranges are bad health scores, the middle of the range must be a good score. This is not a priori true: the middle of the range could be bad too! Absent some external criterion, there's no way for the authors to really figure out what "optimal" might be.

This section has been removed from the revised manuscript, in light of conceptual and textual changes as a result of the inclusion of a qualitative assessment of health.

The authors don't mention anywhere the real take-home message of Figure 6, which is that the HSR metric is hugely more variable than all the rest (look at those standard errors!) and is probably useless as a result.

Please apologize the mistake in error bars for HSR in the original figure, which have been corrected in the revised manuscript. Based on reviewer suggestions, we have removed this panel from figure 8 (originally figure 6).

The discussion of the integrated activity (Figure 7 and lines 320-327) is extremely vague and unclear: what does the integral of the population curve tell us that the population averages of the individual statistics don't? The figure legend claims that the normalized IA better distinguishes the daf-16 replicates from WT (and/or daf-2) than the other measures, but that's not visually obvious from Figure 7, nor is there any quantification.

Addressed in lines 408-410.

Because there is only one value per population, performing statistical analysis on the difference in IA between control and RNAi animals is impossible.

Lines 343-345: "This is why relative assessment, by normalization of individual data to the values of respective control populations, facilitates direct comparison over experiments (Fig 7)." This is an assertion, but isn't really backed up by the data. Figure 7 still shows a ton of replicate-to-replicate variability. How much smaller is the normalized inter-replicate variability compared to un-normalized? (At high enough levels of inter-plate variability, normalization can't really help anymore because you can't even count on the experiment-control pairs to be comparable within a replicate.)

From the bulk of literature on replicability of lifespan experiments in C. elegans, it is clear that large differences in both the absolute (days alive) and relative (effect size) values can be expected. Obviously, more spread is to be expected for longer-lived conditions (as is also generally true in our analysis, cf. ***Figure 9****). For example, based on the GeneAge*

database, loss-of-function mutations in daf-2 can cause an increase of lifespan that ranges from 17 to 179 % [8,9], *compared to wild-type, with various studies reporting mean lifespan of daf-2 animals varying from 20 to 29 days* [7,9,10].

We selected plates for this study based on clear inter-plate variability of lifespan curves (since we wanted to capture such expected variation, as mentioned in ***lines 210-213****. For the reviewer's interest, we have included the coefficient of variation (CV) of absolute and normalized (mean) values (to internal controls) for each of the primary metrics. As can be seen, also in our hands, normalizing reduces (inter-experiment) variation.*

LS	absolute value (mean, in days)				normalized value				CV (abs)	CV (norm)
	EXP I	EXP II	EXP III	EXP IV	EXP I	EXP II	EXP III	EXP IV		
EV	15	12	13	17	NA	NA	NA	NA	**0.21**	**NA**
daf-2 RNAi	26	24	17	29	1.72	1.96	1.26	1.69	**3.05**	**0.17**
daf-16 RNAi	12	10	11	14	0.82	0.82	0.81	0.80	**0.13**	**0.02**

TDH	absolute value (mean, in days)				normalized value				CV (abs)	CV (norm)
	EXP I	EXP II	EXP III	EXP IV	EXP I	EXP II	EXP III	EXP IV		
EV	5	6	6	12	NA	NA	NA	NA	**0.47**	**NA**
daf-2 RNAi	6	8	5	14	1.25	1.36	0.96	1.15	**0.47**	**0.15**
daf-16 RNAi	4	5	5	10	0.85	0.90	0.90	0.83	**0.44**	**0.04**

HR	absolute value (mean)				normalized value				CV (abs)	CV (norm)
	EXP I	EXP II	EXP III	EXP IV	EXP I	EXP II	EXP III	EXP IV		
EV	0.72	0.82	0.83	0.87	NA	NA	NA	NA	**0.08**	**NA**
daf-2 RNAi	0.61	0.74	0.71	0.73	0.85	0.90	0.85	0.84	**0.08**	**0.03**
daf-16 RNAi	0.69	0.87	0.80	0.89	0.96	1.06	0.95	1.02	**0.11**	**0.05**

IA	absolute value				normalized value				CV (abs)	CV (norm)
	EXP I	EXP II	EXP III	EXP IV	EXP I	EXP II	EXP III	EXP IV		
EV	3988	4407	4771	8860	NA	NA	NA	NA	**0.41**	**NA**
daf-2 RNAi	5040	5216	4091	6928	1.26	1.18	0.86	0.78	**0.22**	**0.23**
daf-16 RNAi	2867	4092	3013	5583	0.72	0.93	0.63	0.63	**0.32**	**0.19**

Table R1. Coefficient of variation (CV) is lower for normalized values. *Normalized values, compared to internal control, for the different metrics (LS, TDH, HR and IA) are shown on the right side of the table. Coefficient of variation (CV, the ratio of the standard deviation and the mean) was calculated for absolute (left side of the table) and normalized values, with CV of normalized values being consistently lower.*

Lines 377-379: "In line with literature, this classifies the ‘gerospan’ or ‘extended twiglight’ of daf-2-like interventions as unhealthy". This isn't really consistent with the authors' data, which show that daf-2 has a larger TDH, HS and IA than WT. The HR is smaller for daf-2, and the HSR is so variable as to be useless (as above). Thus across many of the authors own measures daf-2 is perfectly healthy. The "deficiency" of daf-2 is that while it gains many more days of good health vs. WT, it also gains even more days of poor health. Whether or not this is a "good" tradeoff is a

value judgement rather than an empirical fact. Moreover, as per Hahm 2015, under other metrics of health daf-2 behaves quite differently.

Related to the above, lines 384-385 are also an assertion / value judgement masquerading as fact: "interventions that increase health more than they increase longevity [are] a combination of high biological and medical interest". Take for example a condition in which daf-2 individuals are euthanized the second that their activity falls below whatever threshold is defined for "good health". This condition would produce a substantial increase in health with a much more modest increase in lifespan (i.e. this would truncate the "extended twilight" tail of daf-2). Would such an intervention really be better or more medically interesting than a daf-2-like intervention that doesn't involve euthanasia? The fact that lifespan-limiting "interventions" can trivially produce health ratios of 1 suggests that the HR may not really be the most biologically informative measure of "good health". Some careful discussion of what health ratios actually mean (if anything) is in order.

Addressed in lines 329-363 *of the revised manuscript.*

MINOR CONCERNS

Typos or vague / unclear language :

lines 71-75: run-on / complex / overly informal sentence.

Addressed in lines 71-75.

line 139: “day-tot day”

lines 218-221: what is actually being said here?

lines 278-281: likewise unclear.

line 378: “twiglight”

We thank the reviewer for their observations. Due to textual changes in the manuscript, these lines are no longer present.

More care should be taken in referencing:

ref 12, the Lifespan Machine paper, is not particularly germane to the claim on lines 171-173 that "The exact effects of these genetic interventions on the lifespan of C. elegans varies somewhat in high-throughput screens and between different labs".

References have been added to support the complete statement and the text has been adapted accordingly.

The following aren't in the correct format / style:

line 50: Bansal et al.

line 89: Wormbook

*Adjusted in revised manuscript, **lines 49 and 88***

Probably should also cite Herndon 2002, Huang 2004, Pincus 2011, and Zhang 2016 regarding movement decline as a predictor of lifespan (line 52). Likewise, citations for longitudinal analysis of individuals / populations are a bit spotty. Hulme 2010, Pincus 2011, Zhang 2016 would be good to include for the individual case, and both of the Stroustrup papers for populations.

*Adjusted in revised manuscript, **line 58**.*

line 113-115: Citing Hahm 2015 here is deceptive. They showed that spontaneous movement on food is confounded by genotype, compared to maximum velocity off food. But the Hahm results have nothing at all to say about whether stimulated movement (on food) is more or less confounded than spontaneous movement (on food), which is the point that the present authors are attempting

to make. If anything, the original Churgin paper may better support this statement. If this is an important of a point to emphasize, the authors should explain their rationale more carefully.

Agreed, we adjusted this accordingly, ***line 120****.*

Lines 232-234: "Based on these considerations, we opted for the peak activity as the activity value of choice, preferring it over the maximal activity solely based on the fact that the first value is based on more observation points and might therefore be a more accurate representation of true biology." This is not really a mathematically or statistically cogent statement. Calculating a percentile uses all of the input data in the same way that calculating a mean does. It doesn't somehow use "more" data to calculate a percentile and then calculate a mean based off of a subset of the data defined by the percentile.

Addressed in lines 293-296*. It is true that population data are used for calculation of any percentile. However, due to non-normality and variation of the data (cf above), Q99 is a single value at risk of being variable, whereas peak activity has some buffering capacity against outliers. We rephrased this sentence in the revised manuscript, to avoid unnecessary confusion.*

Figure 2: (a) Consider labeling titles/axes for each respective graph to identify which statistic is used to generate the data. (b) Panels B & C for 99th and peak activity appear identical; check whether these graphs have been correctly generated.

Addressed in Figure 2. *Please note that panels B and C indeed look similar (as makes sense based on strong correlation of max and peak data), but contain different values, e.g. clearly visible at the third point in the series which is above 1000 activity units in panel B and under 1000 activity units in panel C.*

Figure 5: Why do daf-2 lifespan estimates decrease with the longest time intervals? Are the animals somehow returning back to where they started such that the difference scores decrease and they register as "dead" incorrectly? This is a more than a little odd...

> ***Addressed in figure 6*** *(previously 5). We must apologize, because this is due to an error in an earlier version of the analysis code. The corrected figure 6 confirms the earlier-reported absence of significant differences between lifespans calculated at 540 seconds vs those of smaller time intervals, and does not show this wrongly-plotted trend anymore.*

Figure 7: It would be helpful to explicitly mention that (a) everything in this figure is normalized to the mean of WT, and (b) IA stands for integrated activity. (Including in the relevant methods section.)

> ***Addressed in figure 9*** *(previously 7).*

Though mentioned in Methods, consider reintroducing the concept of HR/HSR in the Results in greater detail than lines 255-6. These terms are heavily used in the Results and it is a bit be difficult for the reader absent a refresher.

> ***Addressed in lines 352-355***

Table S4: Consider making the second column title for Table S4 “p-value for interaction with experiment”

> *Due to revisions, this table is not included in the manuscript anymore.*

References

[1] Bansal A, Zhu LJ, Yen K, Tissenbaum HA. Uncoupling lifespan and healthspan in *Caenorhabditis elegans* longevity mutants. Proc Natl Acad Sci U S A 2015;112:E277-86. doi:10.1073/pnas.1412192112.

[2] Leiser SF, Jafari G, Primitivo M, Sutphin GL, Dong J, Leonard A, et al. Age-associated vulval integrity is an important marker of nematode healthspan. Age (Omaha) 2016;38:419–31. doi:10.1007/s11357-016-9936-8.

[3] Gelino S, Chang JT, Kumsta C, She X, Davis A, Nguyen C, et al. Intestinal Autophagy Improves Healthspan and Longevity in C. elegans during Dietary Restriction. PLoS Genet 2016;12:e1006135. doi:10.1371/journal.pgen.1006135.

[4] Herndon LA, Schmeissner PJ, Dudaronek JM, Brown PA, Listner KM, Sakano Y, et al. Stochastic and genetic factors influence tissue-specific decline in ageing C. Elegans. Nature 2002;419:808–14. doi:10.1038/nature01135.

[5] Pitt JN, Strait NL, Vayndorf EM, Blue BW, Tran CH, Davis BEM, et al. WormBot, an open-source robotics platform for survival and behavior analysis in C. elegans. GeroScience 2019. doi:10.1007/s11357-019-00124-9.

[6] Petrascheck M, Miller DL. Computational Analysis of Lifespan Experiment Reproducibility. Front Genet 2017;8:92. doi:10.3389/fgene.2017.00092.

[7] Churgin MA, Jung SK, Yu CC, Chen X, Raizen DM, Fang-Yen C. Longitudinal imaging of caenorhabditis elegans in a microfabricated device reveals variation in behavioral decline during aging. Elife 2017;6:e26652. doi:10.7554/eLife.26652.

[8] Hansen M, Hsu AL, Dillin A, Kenyon C. New genes tied to endocrine, metabolic, and dietary regulation of lifespan from a Caenorhabditis elegans genomic RNAi screen. PLoS Genet 2005;1:0119–28. doi:10.1371/journal.pgen.0010017.

[9] Curran SP, Ruvkun G. Lifespan regulation by evolutionarily conserved genes essential for viability. PLoS Genet 2007;3:0479–87. doi:10.1371/journal.pgen.0030056.

[10] Larsen PL, Albert PS, Riddle DL, Hall T. Genes that Regulate Both Development and Longevity in Caenorhabditis elegans. 1995.
